# Morphology of First Zoeal Stage of Four Genera of Alvinocaridid Shrimps from Hydrothermal Vents and Cold Seeps: Implications for Ecology, Larval Biology and Phylogeny

**DOI:** 10.1371/journal.pone.0144657

**Published:** 2015-12-28

**Authors:** Iván Hernández-Ávila, Marie-Anne Cambon-Bonavita, Florence Pradillon

**Affiliations:** 1 Laboratoire Environnement Profond, Institut Français de Recherche pour l’Exploitation de la Mer, CS 10070, 29280 Plouzané, France; 2 Departamento de Ciencias, Unidad de Cursos Básicos, Universidad de Oriente, Margarita Island, Venezuela; 3 Laboratoire de Microbiologie des Environnements Extrêmes, UMR6197 Ifremer, UBO, CNRS, Institut Français de Recherche pour l’Exploitation de la Mer, CS 10070, 29280 Plouzané, France; Universite Pierre et Marie Curie, FRANCE

## Abstract

Alvinocaridid shrimps are endemic species inhabiting hydrothermal vents and/or cold seeps. Although indirect evidences (genetic and lipid markers) suggest that their larval stages disperse widely and support large scale connectivity, larval life and mechanisms underlying dispersal are unknown in alvinocaridids. Here we provide for the first time detailed descriptions of the first larval stage (zoea I) of four alvinocaridid species: *Rimicaris exoculata* and *Mirocaris fortunata* from the Mid-Atlantic Ridge, *Alvinocaris muricola* from the Congo Basin and *Nautilocaris saintlaurentae* from the Western Pacific. The larvae were obtained from onboard hatching of brooding females (either at atmospheric pressure or at habitat pressure in hyperbaric chambers) and from the water column near adult habitats, sampled with plankton pumps or sediment traps. Major characteristics of the alvinocaridid larvae include undeveloped mandible and almost complete absence of setation in the inner margin of the mouth parts and maxillipeds. Although the larvae are very similar between the four species studied, some morphological features could be used for species identification. In addition, undeveloped mouthparts and the large amount of lipid reserves strongly support the occurrence of primary lecithotrophy in the early stage of alvinocaridids. Although lecithotrophy in decapod crustaceans is usually associated with abbreviated larval development, as a mechanism of larval retention, morphological and physiological evidences suggest the occurrence of an extended and lecithotrophic larval stage in the Alvinocarididae. These traits permit the colonization of widely dispersed and fragmented environments of hydrothermal vents and cold seeps. Distribution of larval traits along the phylogenetic reconstruction of the Alvinocarididae and related families suggest that lecithotrophy/planktotrophy and extended/abbreviated development have evolved independently along related families in all potential combinations. However, the Alvinocarididae is the only taxa with a combination of lecithotrophy and extended larval development.

## Introduction

Shrimps of the family Alvinocarididae inhabit deep-waters in the Atlantic, Pacific and Indian Oceans, usually at depths greater than 1000 m [1]. They occur at hydrothermal vents and/or cold-seeps, and could represent the dominant species in these ecosystems, even in some cases forming large aggregations of thousands of individuals per m^2^ [2,3]. Some species such as *Rimicaris exoculata* harbour symbiotic bacteria in their gill chambers and within their guts, which supply nutrients to the shrimp in a complex mutualistic association [4], whereas other species depend on grazing on chemoautotrophic bacteria and detritus in their adult stage [2].

However, populations of these shrimps exhibit high genetic connectivity along the Mid-Atlantic Ridge [5], between different vent and cold seeps systems in the Atlantic [6] and in vents systems of the Western Pacific and Indian Oceans [7,8]. This suggests a notable ability for dispersion and migration in widely spaced habitats. Juveniles and adult stages of alvinocaridids inhabit close to the vents and cold seeps due to their dependence on bacterial-detrital grazing or to their need to supply their symbiotic bacteria with reduced compounds, limiting adult migration [4,6]. Colonization of new habitats and connectivity along their geographic range must be promoted by larval forms. However, information about larval stages is scarce, limited to reports of post-larvae in plankton samples [9,10], estimations of larval development mode based on egg size [11], and on board observations of larvae hatched from brooding females [10, 12–17]. In general, published data on larval forms of alvinocaridid shrimps include only brief descriptions or some illustrations, but without clues about important larval traits, differences between species or comparison with other caridean shrimps.

As other carideans, brooding females of alvinocaridids maintain their eggs below the abdomen during the embryonic development until the hatching, which is followed by a planktonic larval stage [15,17,18]. Larval history appears to occur in the water column, where, after a series of molts, post-larval stages recruit in the benthic system of hydrothermal vents and cold seeps. However no information is available about the duration, dispersion or development of larvae, and no field samples of larval stages have been reported at the present (except briefly by Miyake [19]). The absence of larval descriptions in this group is a gap to be resolved, because it could help to identify larvae in the plankton and elucidate mechanisms of dispersion and larval history. Also larval morphology can bring important cues about the early life history of the species [20] and provide useful morphological features to help interpreting both phylogenies [21,22] and ecology [23,24]. In this paper we describe the first larval stage of four genera of Alvinocarididae obtained from hatching of gravid females and some larvae identified from field plankton samples.

## Material and Methods

### Collection and preparation of larval stages

Brooding females of *Rimicaris exoculata*, *Mirocaris fortunata*, *Nautilocaris saintlaurentae* and *Alvinocaris muricola* were collected from hydrothermal vents on the Mid-Atlantic Ridge (*R*. *exoculata* and *M*. *fortunata*), in the Western Pacific (*N*. *saintlaurentae*), and at cold seeps of the Congo Basin (*A*. *muricola*) (Table 1). Shrimps were collected with a suction sampler connected to the submersible (Nautile or ROV VICTOR 6000), and in most of cases, were brought on board the research vessel (N/O *L’Atalante* or *Pourquoi Pas*?) without pressure compensation. Adult females with eggs in advanced stage of development were examined, and in some of them, eggs had hatched (maybe due to recovery stress). After hatching, active larvae, rests of eggs and no-motile larvae were fixed in 4% buffered formalin, 10% glutaraldehyde, or 96% ethanol. Adult females were preserved and identified by external morphology [25,26] and by DNA mitochondrial COI sequence (for *N*. *saintlaurentae*).

**Table 1:** Cruise and sample information for the larvae herein studied.

Species	Sample	Location	Site	Coordinates	Cruise	Depth (m)	Date	Sampling gear
*R*. *exoculata*	Lab. hatched	MAR	Logatchev	14°45'N; 44°57'W	Serpentine	3037	March 2007	Suction sampler
*R*. *exoculata*	Lab. hatched	MAR	TAG	26°08' N; 44°49' W	BICOSE	3635	January 2014	Suction sampler
*R*. *exoculata*	Lab. hatched, with habitat pressure	MAR	TAG	26°08' N; 44°49' W	BICOSE	3635	February 2014	Suction sampler, pressure compensation
*R*. *exoculata*	Plankton samples	MAR	TAG	26°08' N; 44°49' W	BICOSE	3637	February 2014	Plankton pump
*A*. *muricola*	Lab. hatched	Gulf of Guinea	pockmark Regab	05°48'S; 09°42' W	Biozaïre 2	3150	November 2001	Suction sampler
*A*. *muricola*	Plankton samples	Gulf of Guinea	pockmark Regab	5°48' N; 9°42' W	Congolobe	3150	January 2012	Sediment trap
*M*. *fortunata*	Lab. hatched	MAR	Lucky Strike	37°17' N; 32°17' W	Momarsat	1739	September 2013	Suction sampler
*N*. *saintlaurentae*	Lab. hatched	Wallis and Futuna	Fatu Kapa [30]	14°N; 177°'W	Futuna 3	1554	June 2012	Suction sampler

For the laboratory reared larvae, sampling information refers to the brooding female.

Brooding females of *R*. *exoculata* collected during the BICOSE cruise in January 2014 at the TAG site of the MAR were brought on board with pressure compensation in the PERISCOP chamber [27] and were maintained in the BALIST chamber [28] at 300 bars and 5°C during 24 hours. Hatching occurred in the pressure chamber and active larvae were separated after a short period of decompression. Larvae were maintained in the PICCEL chamber [29] at 300 bars and 8°C during 96 hours. Larvae hatched at habitat pressure were compared with the larvae hatched at atmospheric pressure in order to assess the potential effects of hatching at low pressure on larval morphology.

Additionally, larvae collected in deep-water plankton samples from different locations were studied. Larvae of alvinocaridid shrimps (6 specimens) from the Congo Basin were collected using sediment traps deployed at 3150 m depth at the cold seep site REGAB in the Congo Basin. On the MAR, a larval pump SALSA (Serial Autonomous Larval Sampler- Ifremer) deployed at the TAG site at 3637 m captured 6 specimens of alvinocaridid larvae, 3 of which are included in the present study (Table 1). The morphology of these larvae was compared with the larvae obtained from hatching on board for species identification.

For larvae obtained from on board hatching, from 8 to 15 larvae of each species were fully dissected, and mounted on slides for observations and drawings with a Leica-Leitz microscope with camera lucida. Measurement of total length (TL) and carapace length (CL) were performed for each specimen and variation in the number of setae and spines were noted, including variation between the appendices of the left and right side in the same specimen. For the larvae obtained from field samples, some specimens were dissected for observation and drawings. For general considerations during dissection, mounting, description and illustration we followed Clark *et al*. [31].

Some specimens from both laboratory hatching and field samples were prepared for Scanning Electron Microscopy (SEM) analyses. Specimens previously fixed in 2.5% glutaraldehyde (seawater) or in 4% formalin were washed (3 times, 5 min each) in distilled water and post-fixed in 0.8% osmium tetroxide (OsO_4_) for 1 h. Later the samples were washed 3 times in water and fixed again in OsO_4_ for 1h, rinsed (6 times, 5 min each) in distillated water and dehydrated in graded ethanol series (10, 25, 35, 50, 65, 70, 80, 90, 96, 100%, 5 min each, 3 times for the last 5 grades). Samples were submitted to a critical point (Leica EM CPD300), sputter in gold, and observed in SEM.

### DNA extraction, amplification, sequencing and phylogenetic reconstruction

Extractions were performed from whole larvae, or 1–2 pleopods for the adult specimens collected at MAR and Western Pacific hydrothermal vents, using the CTAB method (cetyl trimethyl ammonium bromide [32]). Amplifications of the COI gene were performed in a 50 μL solution of 1X reaction buffer, 2 mM MgCl_2_, 0.25 mM dNTPs, 0.6 mM of each primer (LCOI1490 and HCOI2198 [33]), 1.25 U *Taq* polymerase and 1–4 μL extracted DNA (depending on the DNA concentration). We performed 35 cycles of amplification with an annealing temperature of 52°C. Similarly, amplifications of the 18S rRNA gene were performed in a 50 μL solution of 1X reaction buffer, 2 mM MgCl_2_, 0.4 mM dNTPs, 0.5 mM of each primer (18S1 Forward and 1498 Reverse [34]), 1.25 U *Taq* polymerase and 1 μL extracted DNA. We performed 30 cycles of amplification with an annealing temperature of 51°C. All PCR amplifications were conducted on a GeneAmp PCR system 9700 (Applied Biosystems). PCR products were purified and sequenced at Macrogen, Inc. (Netherlands) using the amplification primers for COI and for the 18S gene the following primers: 18S-3F, 18S-bi, 18S-1F, 18S-5r, 18S-1373F, 18S-505r [34–36].

Other sequences included in the phylogenetic reconstructions were obtained from different studies, mostly related with phylogeny and population genetics on alvinocaridids [5–7,37–41], and available in genbank (S1 Table and S2 Table). For the phylogenetic reconstructions, the sequences were aligned using MUSCLE [42]. Phylogenetic trees were constructed using Bayesian Inference (BI) in BEAST version 1.8 [43]. Configurations of the evolutionary model for each data set were selected according to the best-fit obtained from Model Generator [44]. The selected model of nucleotide substitution for the COI was HKY + I + G, considering a speciation Yule process. Whereas for the 18S, the best-fit model was HKY + G, with a speciation Yule process. 20^6^ generations trees with sampling each 1000 generations were performed, the first 25% of the trees were discarded, the rest of the trees were summarized using Treeannotator. Robustness of the inferred trees was evaluated using posterior probabilities. For the COI phylogenetic reconstruction, few sequences (8 of the 201 COI sequences analyzed) obtained from Genbank were discarded due inconsistences (outgroup sorting, and non-monophyletic distribution of the same species) observed in early analyses.

### Relationship of the DWCC phylogenetic reconstruction and larval traits

In order to interpret larval morphology traits in an ecological and evolutionary context, literature data on larval traits in related families were also examined. 18S rRNA nuclear gene was used to reconstruct a phylogeny of alvinocaridid shrimps and related families. According to Braken *et al*. [39], Li *et al*. [40] and Aznar-Cormano *et al*. [41] Alvinocarididae are closely related to 7 other families of mainly deep-water Caridea (Oplophoridae, Nematocarcinidae, Agostocarididae, Campylonotidae, Pasiphaeidae, and Psalidopodidae, with some differences between authors), herein mentioned as deep-water caridean clade (DWCC, although these are not the only deep-water families and include a few shallow-water taxa). Sequences of these major taxa were obtained from GenBank, including those used in the phylogenetic analyses of the former studies [39–41] and others as well (S2 Table). Moreover we obtained new sequences of the 18S gene for *R*. *chacei*, *M*. *fortunata* and *N*. *saintlaurentae*. Rather than bringing a new phylogenetic proposal for the group, our intent was to set an evolutionary framework to analyze the occurrence of larval traits in the DWCC.

In order to compare the phylogenetic relationships along the DWCC and the occurrence of larval traits, we sorted the larval information related to the species considered in the phylogenetic reconstruction. We considered general traits of the larval morphology as indicators of some aspects of the early larval biology. Undeveloped mouth parts (absence of incisive and molar processes in the mandible, and lack or poor setation and spination in the maxillule and maxilla) was considered as indicator of primary lecithotrophy, because the larvae is not able to take external source of food and so relies on its lipid reserves [45]. On the contrary, larvae with developed mouth parts were categorized as planktotrophic, because the larvae have the ability to capture and ingest food when it becomes available. However some planktotrophic larvae could also hatch with large amount of lipid reserves (usually accomplished by large eggs > 1 mm), which could enhance their nutritional flexibility [20]. Unfortunately the descriptions of lipid reserves in the group are scarce and range between just “oil drops” to isotopic analysis, and a more accurate classification distinguishing facultative lecithotrophic from planktotrophic larvae was limited in some cases.

Moreover the presence of pereiopods and/or pleopods in hatching larvae was considered as a trait related with abbreviated development [20,45]. In general, decapod crustacean larvae hatch without pereiopods or pleopods. The development of these structures occurs usually as the larvae molt into more advanced stages. The occurrence of these structures in the first stage suggests advanced development and reduction of the larval instars. For some species considered in our phylogenetic analysis, no information is available regarding the larval development. In these cases, traits were inferred from other species of the same genus with existing knowledge about larval traits (but without genetic data that prevented their inclusion in our dataset) (S2 Table). Although larvae of the same genus usually show interspecific differences in spines and setae, there is no evidence of variation within genera for the major larval traits herein considered (i.e. mouth parts development, occurrence of pereiopods and pleopods) in the DWCC.

### Ethics statement

No specific permissions were required to collect the samples used here during the Serpentine and BICOSE cruises (international waters). During the cruises Biozaïre 2 and Congolobe, samples were collected with permission from the Gabon Government. Samples were collected during the cruise MOMARSAT with permission of the Azores Regional Government, Portugal, and during the Futuna cruise with permission of the French Government. The study did not involve endangered or protected species.

## Results

### Description of first larval stage in alvinocaridids shrimps

#### *Rimicaris exoculata* Williams and Rona, 1986

**Fig 1:**
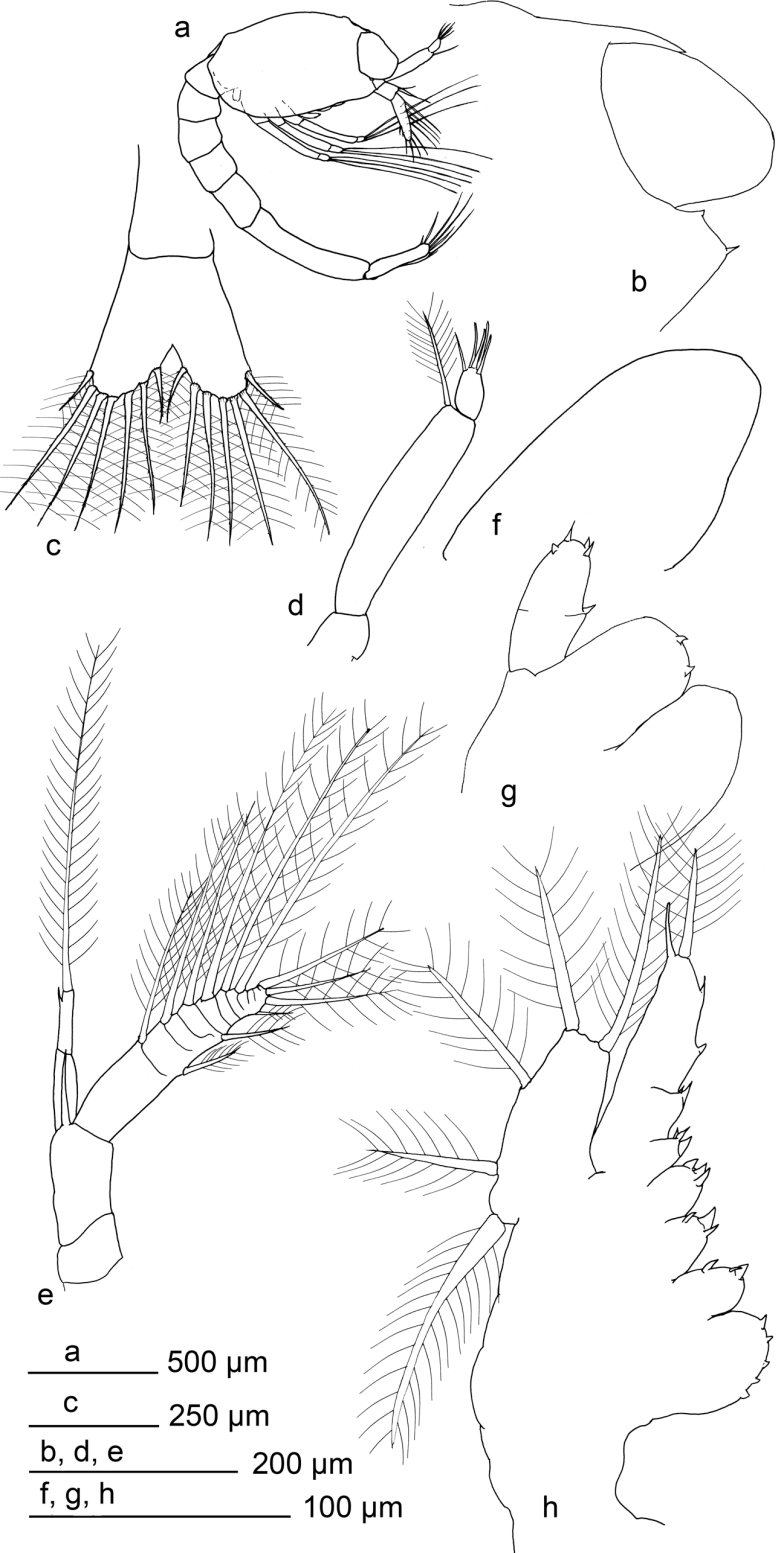
*Rimicaris exoculata* zoea I. a) habitus, b) distal section of carapace, c) telson, d) antennule, e) antennae, f) mandible, g) maxillule, h) maxilla.

**Fig 2:**
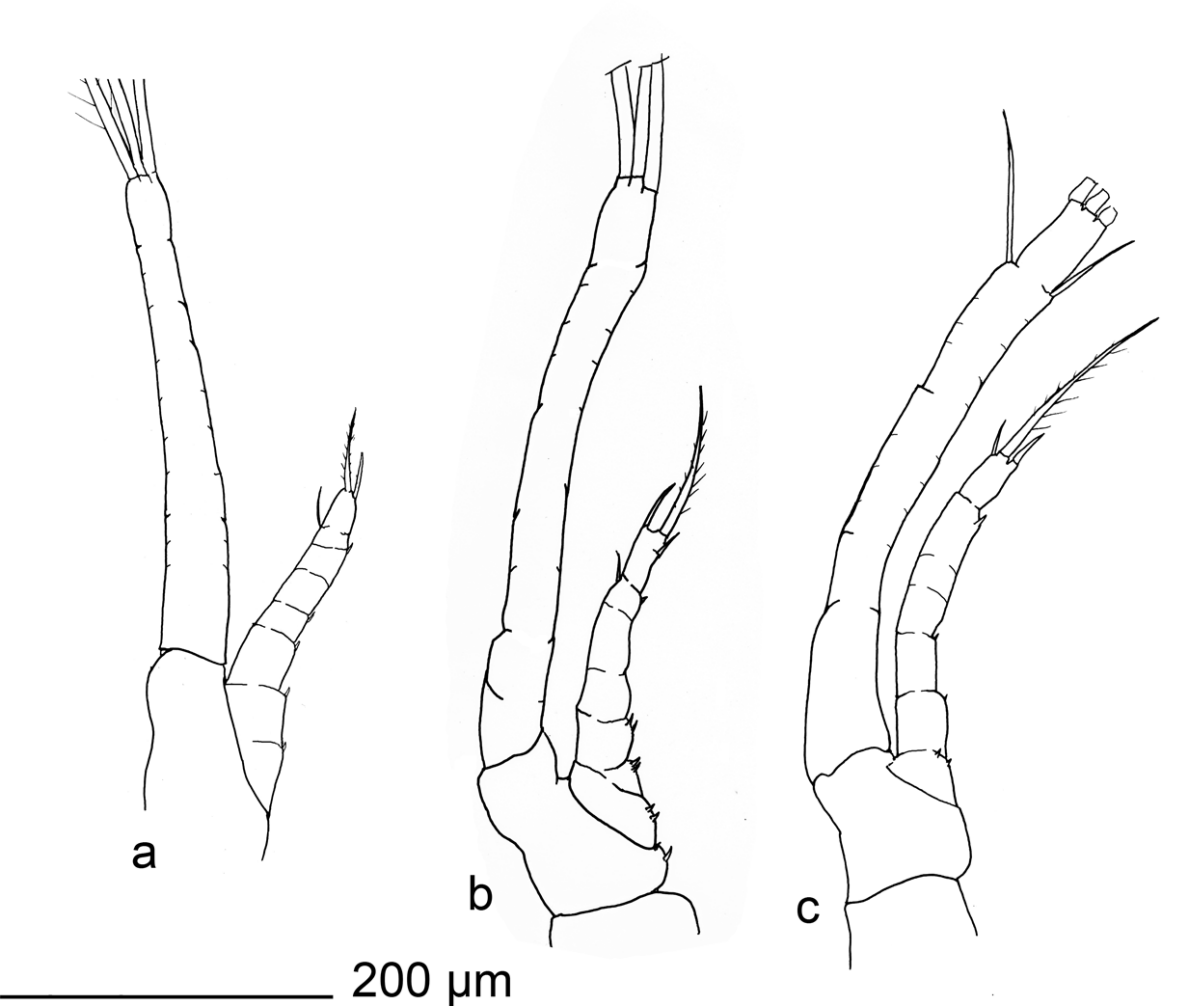
*Rimicaris exoculata* zoea I. maxilipeds, a) first, b) second, c) third.

**Fig 3:**
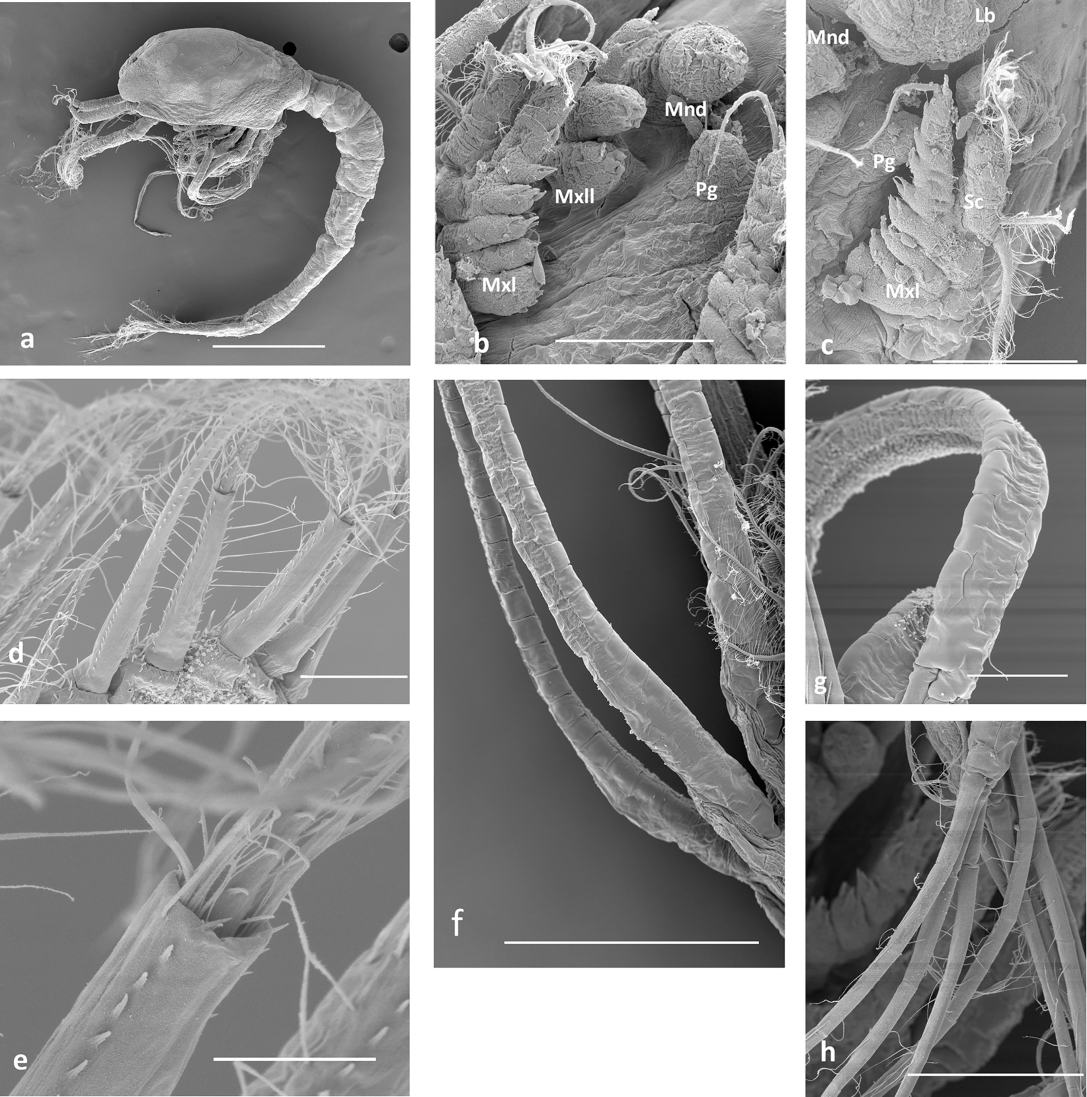
SEM details in some alvinocaridid zoea I larvae. A.- *Rimicaris exoculata*, lateral view; b, c.- *R*. *exoculata*, mouth parts; d, e.- *R*. *exoculata*, plumodenticulate seta of telson; f.- *Mirocaris fortunata*, exopods of 1–3 maxillipeds; g, h.- *R*. *exoculata*, exopod of 2nd maxilliped and distal setae. Mnd, mandibule; Mxll, maxillule; Mxl, maxilla; Pg, paragnathe; Lb, labrum.

**Mid-Atlantic Ridge (Figs 1, 2, 3A–3D, 3G and 3H).** Dimensions: LC = 0.61±0.06 mm; LT = 2.41±0.12 mm.

Carapace with tiny rostrum, sharp but hidden between the eyes, reaching around a half of the eyes length. Eyes sessile. A small anterior-dorsal hump present. Pterisgostomial spine present, followed by small sub-ocular spine. Posterior margin bilobulated in dorsal view. Antennulae uniramous, peduncle unsegmented with 1 distal setulose setae. Distal segment (outer flagellum) with 0–1 setae simple and 3–5 aesthetascs. Basal segment of the antennae (peduncle) unsegmented, armed with a large spine inserted near to the endopod, and approximately 0.6 times the length of the endopod. Endopod bi-articulated, second article with subdistal spine, absent in some specimens, and large setulose setae. Exopod distally segmented and armed with large setae (setulosae), 7 in the inner margin, 3 distally inserted and 2 smaller in the outer margin.

Labrum and paragnathes present, thumb-like. Mandible thumb-like, unsegmented. Incisive and molar processes absents, 0–3 small distal spines. Palp absent. Maxillule with 0–4 spines in coxal endite. Basal endite with 2–3 spines. Endopod composed by two segments, with mesial spine in the inner margin and 2–4 distal spines. Maxilla with endite coxal and basal bilobulated, coxal endite with 3–8 spines in the first lobule and 1–3 spine in the second. Basal endite with 2–4 spines in the first lobule and 1–3 spines in the second. Endopod unsegmented, with two proximal small lobes armed by 1–3 spines and 1–2 spines respectively. Followed by 2 subdistal spines, one distal spine in the outer margin and distal setulose setae 2–5 times the size of the distal spine. Margin of the scaphognathite with 5 marginal setulose setae.

Maxillipeds 1–3 similar in shape. Endopod with irregular segmentation, between 5–6 articles. Distal article with 1–3 plumodenticulate setae, occasionally with additional spine. Inner margin of endopod with 2–6 disperse, small spinules, sometimes with small subdistal setae. Exopod with superficial and incomplete segmentation, with a shallow furrow-like surface in the inner face and flat outer surface. Distally the exopod is armed with 3 large setulose setae. Pereiopod only as rudimentary bud, without segmentation, unarmed.

Abdomen with 6 somites, first segment overlap with the carapace. Last segment larger than others, thinner and dorsally compressed. Setae and spines absent. Ventral humps present in the 1–5 somites, pleopods and uropods absents. Telson bilobulated, distal margin with 7 plumodenticulate setae in each lobe.

Remarks: The larvae hatched in the BALIST chamber and maintained at 300 bars during 96 h did not show differences with the specimens hatched at atmospheric pressure. Although some variation occurs between specimens in the number of spines on the mouth parts and on the maxillipeds, their range completely overlap between the larvae hatched at atmospheric pressure or at habitat pressure. Hatching at atmospheric pressure does not appear to have an effect on the morphology of the larvae of *R*. *exoculata* and we assume that larvae of other species obtained after hatching at atmospheric pressure reflect then the morphology of those at their habitats.

#### *Mirocaris fortunata* (Martin and Christiansen, 1995)

**Fig 4:**
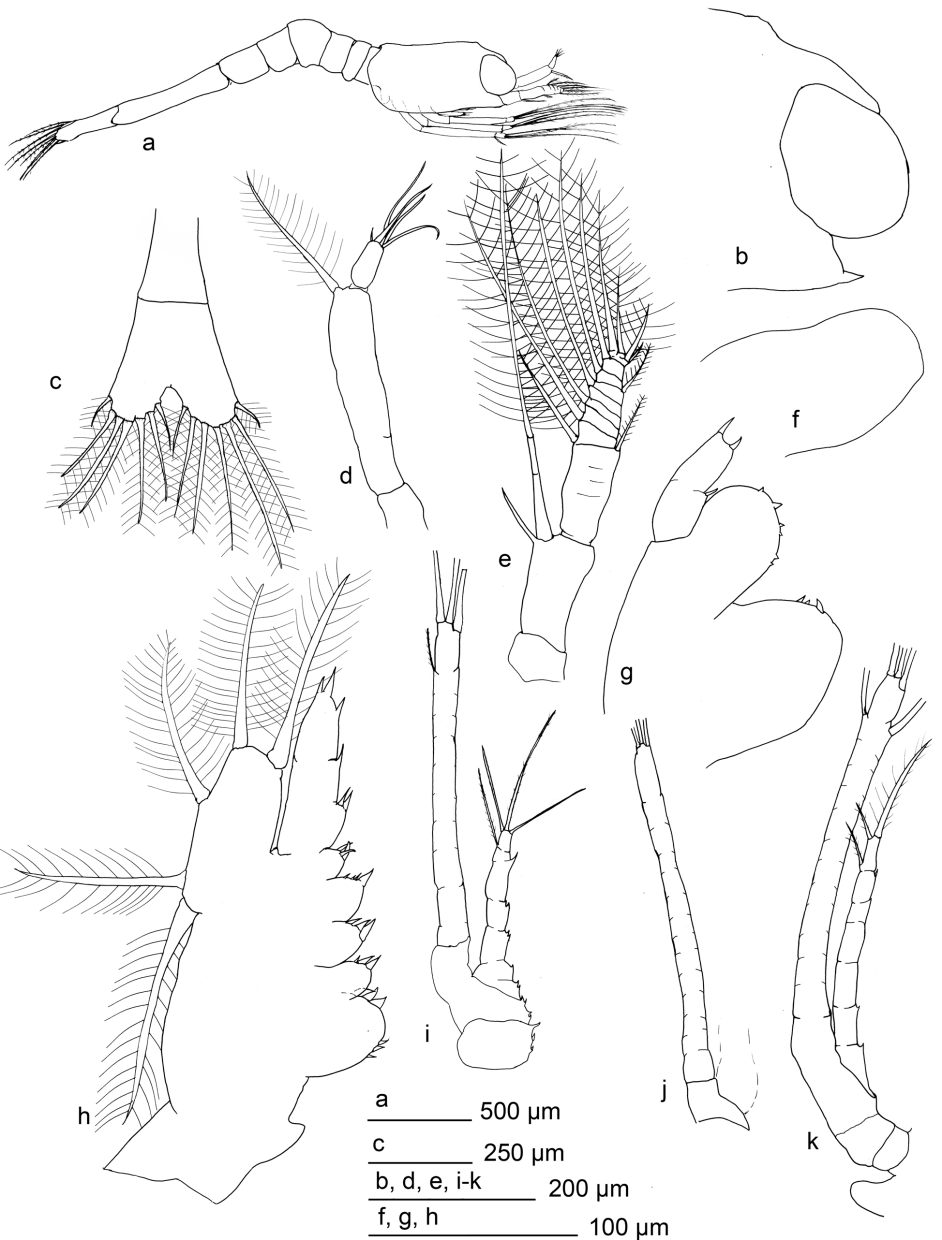
*Mirocaris fortunata* zoea I. a) habitus, b) distal section of carapace, c) telson, d) antennule, e) antennae, f) mandible, g) maxillule, h) maxilla, i-k) first to third maxillipeds respectively.

**Mid-Atlantic Ridge (Figs 3F and 4).** Dimentions: LC = 0.56±0.027 mm; LT = 2.32±0.08 mm.

Carapace with tiny rostrum, sharp but hidden between the eyes, reaching around a half of the eyes length. Eyes sessile. A small anterior-dorsal hump present. Pterisgostomial spine present. Posterior lateral margin bilobulated in dorsal view. Antennulae uniramous, peduncle unsegmented, setulose setae inserted distally. Distal joint (outer flagellum) with 3–5 aesthetascs and, eventually, 1 setae. Antennae with basal segment (peduncle) unsegmented, armed with a distal spine 0.6 times the size of the endopod. Endopod bi-articulated, second article with tiny subdistal spine, sometimes absent, and large setulose setae. Exopod distally segmented and armed with large setae (setulosae), 7 in the inner margin, 2–3 distally inserted and 2 smaller in the outer margin.

Mandible thumb-like, unsegmented. Incisive and molar processes absents, unarmed or with 1–2 small spines. Palp absent. Labrum and paragnathes present, thumb-like, unarmed. Maxillule with coxal endite with 2–4 distal spines. Basal endite with 3–6 spines, some minute. Endopod composed by two segments, with mesial spine in the inner margin and 2 distal spines. Maxilla with two lobules in coxal and basal endites. Coxal endite with 5–6 spines in the first lobule and 2–3 spines in the second. Basal endite with 3 and 4 spines in each lobule. Endopod unsegmented, with two proximal small lobes armed with 3 and 2 spines respectively, followed by 2 subdistal spines and two distal spines. Margin of the scaphognathite with 5 marginal setulose setae.

Maxillipeds 1–3 similar in shape. Endopod with segmentation irregular, uncomplete. Subdistal setae present followed by 2–3 distal setae, irregular in size, setulose. Inner margin of endopod with 5–6 disperse, small spinules. The first maxilliped shows up to 13 spines between the margin of the endopod and coxal-basal margin. Exopod with superficial incomplete segmentation, with 3 large setulose setae distally inserted. Inner and outer faces of exopod show a flat surface, inner also with small furrow. Some specimens also with 1–2 thin sub-distal setae. Pereiopods only as rudimentary bud, without segmentation, unarmed.

Abdomen with 6 somites, first segment overlap with the carapace. Last somite larger and laterally thinner. Setae and spines absent. Ventral humps present in the 1–5 somites, pleopods and uropods absent. Telson bilobulated, distal margin with 7 plumodenticulate setae in each lobe.

#### *Nautilocaris saintlaurentae* Komai & Segonzac, 2004

**Fig 5:**
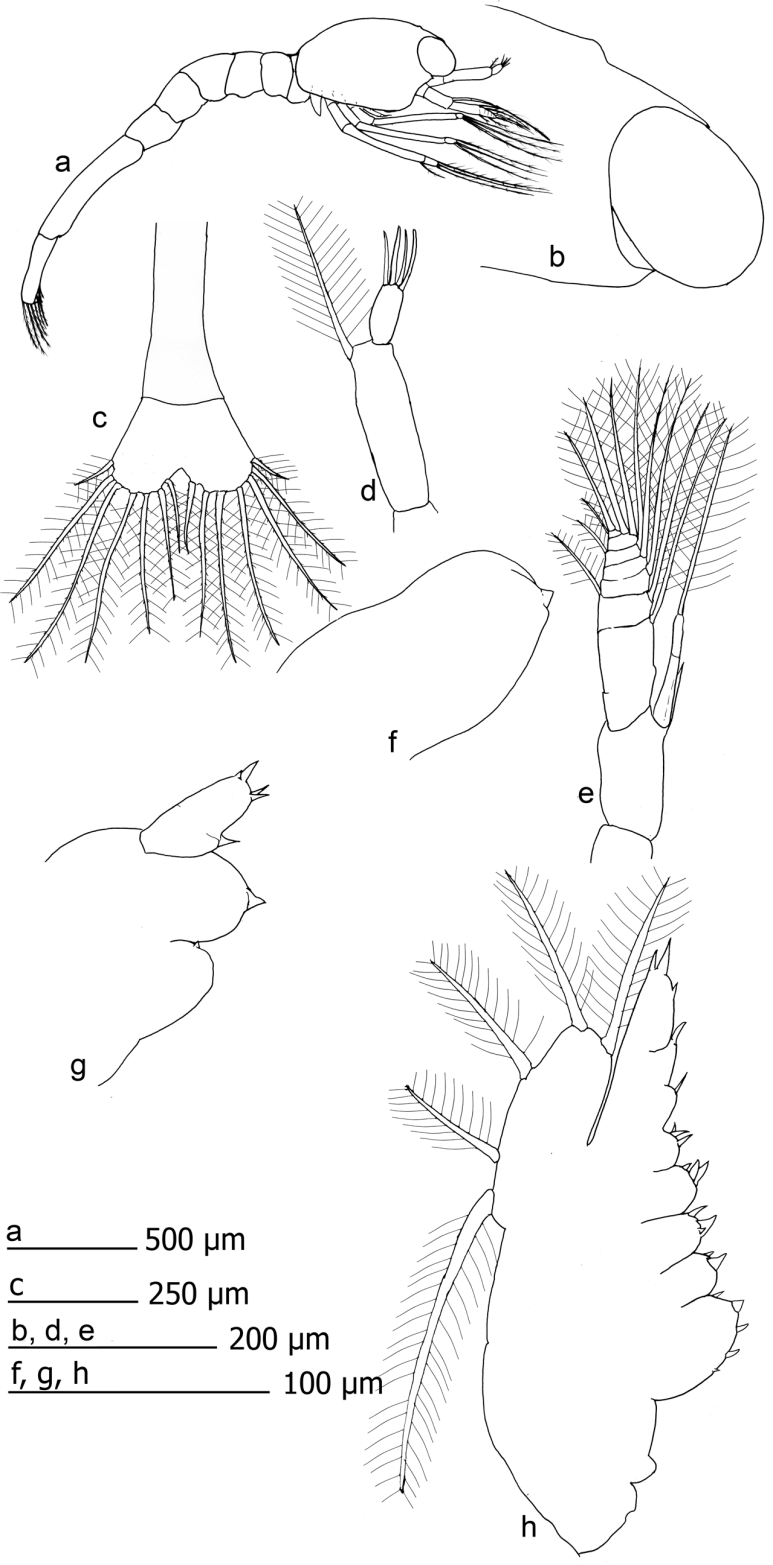
*Nautilocaris saintlaurentae* zoea I. a) habitus, b) distal section of carapace, c) telson, d) antennule, e) antenna, f) mandible, g) maxillule, h) maxilla.

**Fig 6:**
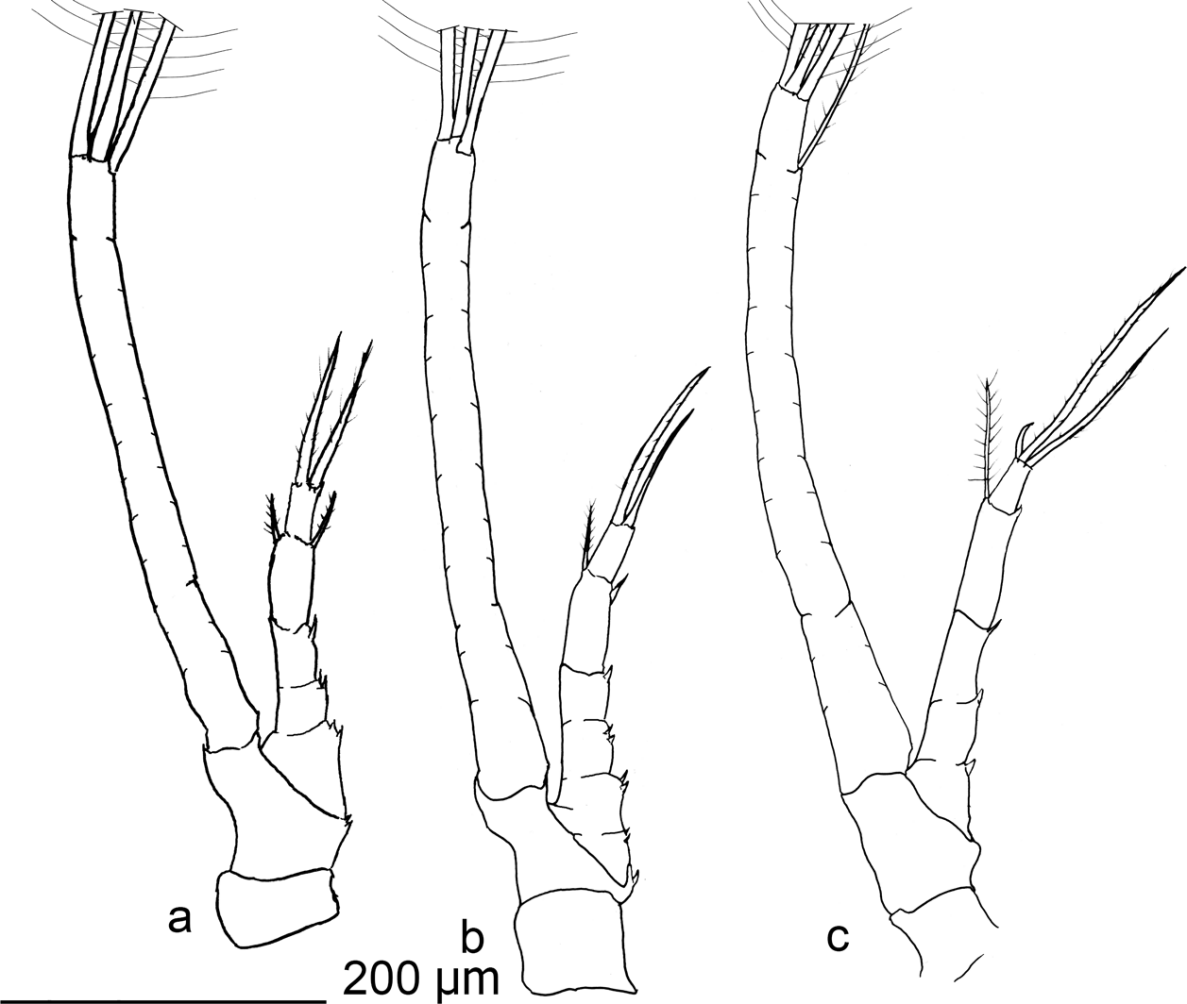
*Nautilocaris saintlaurentae* zoea I. maxillipeds, a-c) first to third respectively.

**Western Pacific (Figs 5 and 6).** Dimensions: LC = 0.58±0.004 mm; LT = 2.20±0.01 mm.

Carapace armed with tiny rostrum, sharp but hidden between the eyes, reaching around a half of the eyes length. Eyes sessile. A small anterior-dorsal hump present. Pterisgostomial spine present. Posterior margin laterally bilobulated. Antennulae uniramous, peduncle unsegmented, setulose setae inserted close to distal joint. Distal segment (outer flagellum) with 3–5 aesthetascs. Basal segment of the antennae (peduncle) unsegmented, with large spine inserted near to the endopod (0.6–1.1 times the size of the endopod). Endopod bi-articulated, second article usually with small subdistal spine and large setulose setae. Exodop distally segmented and armed with large setae (setulosae), 6–7 in the inner margin, 2–3 distally inserted, and 2 smaller in the outer margin.

Mandible thumb-like, unsegmented. Incisive and molar processes absents, unarmed or with 1–2 small spines. Palp absent. Labrum and paragnathes present, thumb-like, unarmed. Maxillule with coxal endite with 0–5 small spines. Basal endite with 1–4 spines, some minute. Endopod composed by two segments, with 1–2 mesial spines in the inner margin and 2–4 distal spines. Maxilla with endite coxal and basal bilobulated, coxal endite with 4–8 spines in the first lobule and 2–3 spines in the second. Basal endite with 2–3 spines in the first lobule and 2–4 in the second. Endopod unsegmented, with two proximal small lobes armed with 1–3 and 1–2 spines respectively, followed by 2 subdistal spines. Distal margin with two spines, a single spine in some specimens. Margin of the scaphognathite with 5 setulose setae.

Maxillipeds 1–3 similar in shape. Endopod with segmentation irregular, 3–6 joints. Distal joint with 2–3 setae, and 1–2 small subdistal setae, all setulose. Inner margin of endopod with 0–8 disperse, small spinules. Exopod show superficial segmentation at lateral sides and flat inner and outer faces. Small furrow also on the inner face of expodod. Three large setulose setae inserted distally. Some specimens also with 1–2 sub-distal setae. Pereiopods only as rudimentary bud, unarmed.

Abdomen with 6 somites, first segment overlap with the carapace. Last somite larger and laterally thinner. Setae and spines absent. Ventral humps present in the 1–5 somites, pleopods and uropods absent. Telson bilobulated, distal margin with 7 plumodenticulate setae in each lobe.

#### *Alvinocaris muricola* Williams, 1988

**Fig 7:**
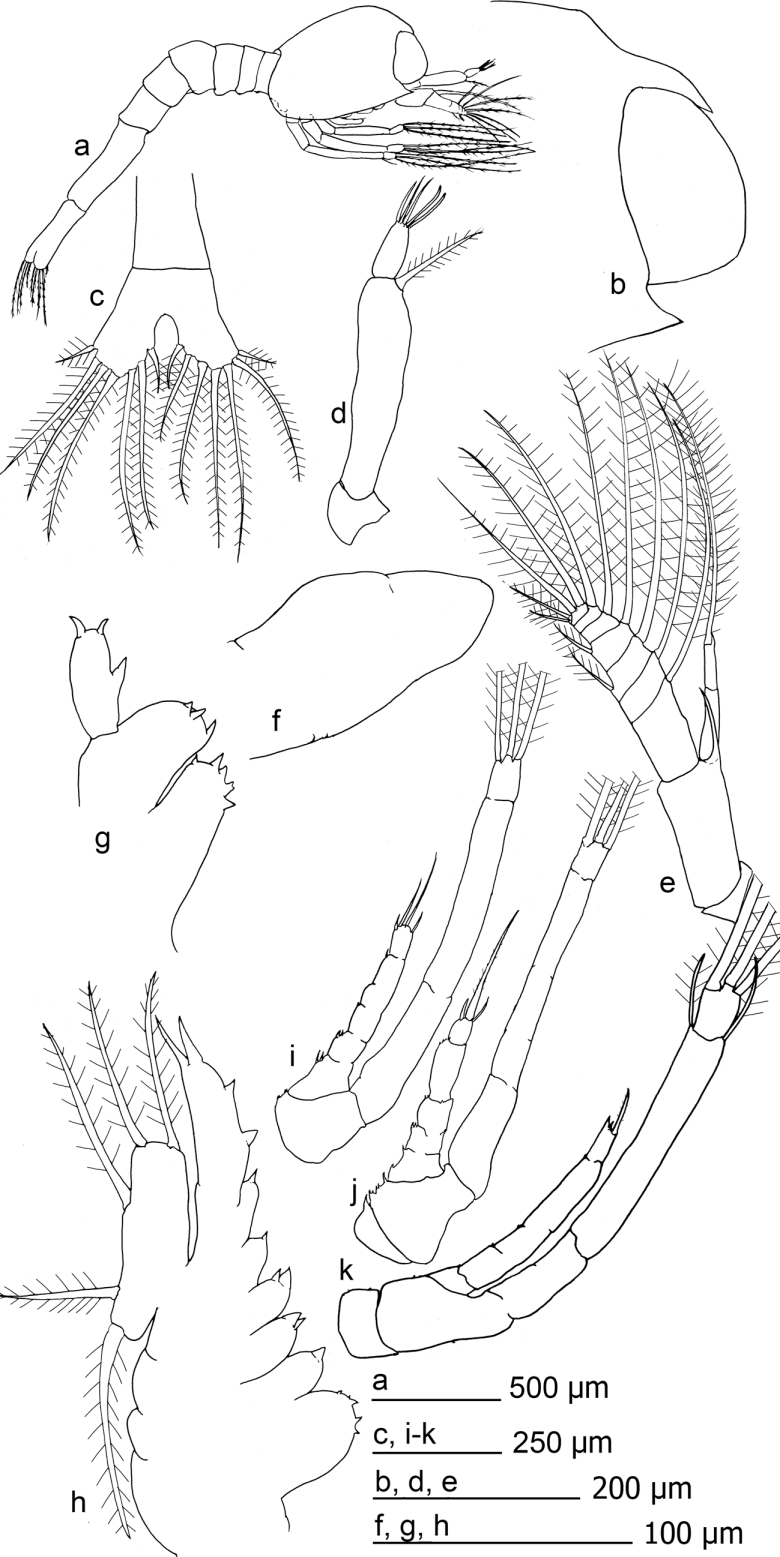
*Alvinocaris muricola* zoea I. a) habitus, b) distal section of carapace, c) telson, d) antennule, e) antenna, f) mandible, g) maxillule, h) maxilla, i-k) first to third maxillipeds respectively.

**Eastern Atlantic, Congo Basin (Fig 7).** Dimensions: LC = 0.57±0.02 mm; LT = 2.13±0.07 mm.

Carapace with tiny rostrum, sharp but hidden between the eyes, reaching around a half of the eyes length. Eyes sessile. A small anterior-dorsal hump present. Pterisgostomial spine present. Posterior margin laterally bilobulated. Antennulae uniramous, peduncle unsegmented, large setulose setae inserted close to distal joint. Distal segment (outer flagellum) with 5 aesthetascs. Antennae with basal segment (peduncle) unsegmented, armed by a large spine inserted near to the endopod. Endopod bi-articulated, second article with distal spine and large setulose setae. Exopod distally segmented and armed with large setae (setulosae), 6 in the inner margin, 2 large and 2 small setae distally inserted, 2 small setae inserted in the outer margin.

Mandible thumb-like, unsegmented. Incisive and molar processes absents, unarmed. Palp absent. Labrum and paragnathes present, thumb-like, unarmed. Maxillule with coxal endite with 5 strong spines, one bifid. Basal endite with 3 spines. Endopod composed by single segment, with mesial spine in the inner margin and 2 curved spines distally. Maxilla with coxal and basal endites bilobulated, coxal endite with 5 small and 1 large spines in each lobule, and basal endite with 2 spines in each lobule. Endopod unsegmented, with two proximal lobes armed with 1 spine each followed by 2 subdistal spines, one distal small and thick setae (setulose) and distal spine. Margin of the scaphognathite with 5 setulose setae.

Maxillipeds 1–3 similar in shape. Endopod with segmentation irregular, composed by 4–8 joints. Distal joint with 1 large and 2–3 small setae. Inner margin of endopod with 6–8 disperse, small spinules and 4–5 spinules in the basis. Exopod superficially segmented in lateral sides, distally armed 3 large setulose setae and occasionally with 1–2 small sub-distal setae. Inner and outer faces of the endopod flat, with small furrow in the inner surface. Pereiopods only as rudimentary bud, without segmentation, unarmed.

Abdomen with 6 somites, first segment overlaps with the carapace. Last somite larger and laterally thinner. Setae and spines absent. Ventral humps present in the 1–5 somites, pleopods and uropods absent. Telson bilobulated, distal margin with 7 plumodenticulate setae in each lobe.

### Morphological identification of alvinocaridid larvae collected by plankton samplers

The larvae collected with the plankton pump on the MAR show the same morphological features as the larvae hatched from *R*. *exoculata* females onboard. These characters include the setae at the tip of the endopod of the maxilla and the small subocular spine, which are distinct from other species herein studied. No other species except *R*. *exoculata* exhibit a subocular spine in the carapace, although this small spine was, rarely, absent in some *R*. *exoculata* specimens. Moreover the setae at the tip of the endopod of the maxilla is present in *R*. *exoculata* and *A*. *muricola*, but in the latter species, it is variable in shape from a spine to a small and thick setae with few setulae. Other species, *M*. *fortunata* and *N*. *saintlaurentae* only show two spines at the tip of the endopod.

The shrimp larvae collected with the sediment traps in the Congo Basin shows similar characters as those of specimens of *A*. *muricola* that hatched onboard. Although all alvinocaridid species herein studied are very similar to each other and the knowledge of alvinocaridid larval morphological variation is still low, a thick short setae at the tip of the endopod of the maxilla was observed only in *A*. *muricola* and in the specimens collected with sediment traps. Moreover *A*. *muricola* is the only alvinocaridid species in the Congo cold seeps. The other species with a distal setae in the maxilla endopod, *R*. *exoculata*, shows a long setae and a large spine besides, and also usually a small subocular spine, which is absent in *A*. *muricola*.

### Molecular identification of alvinocaridid larvae

**Fig 8:**
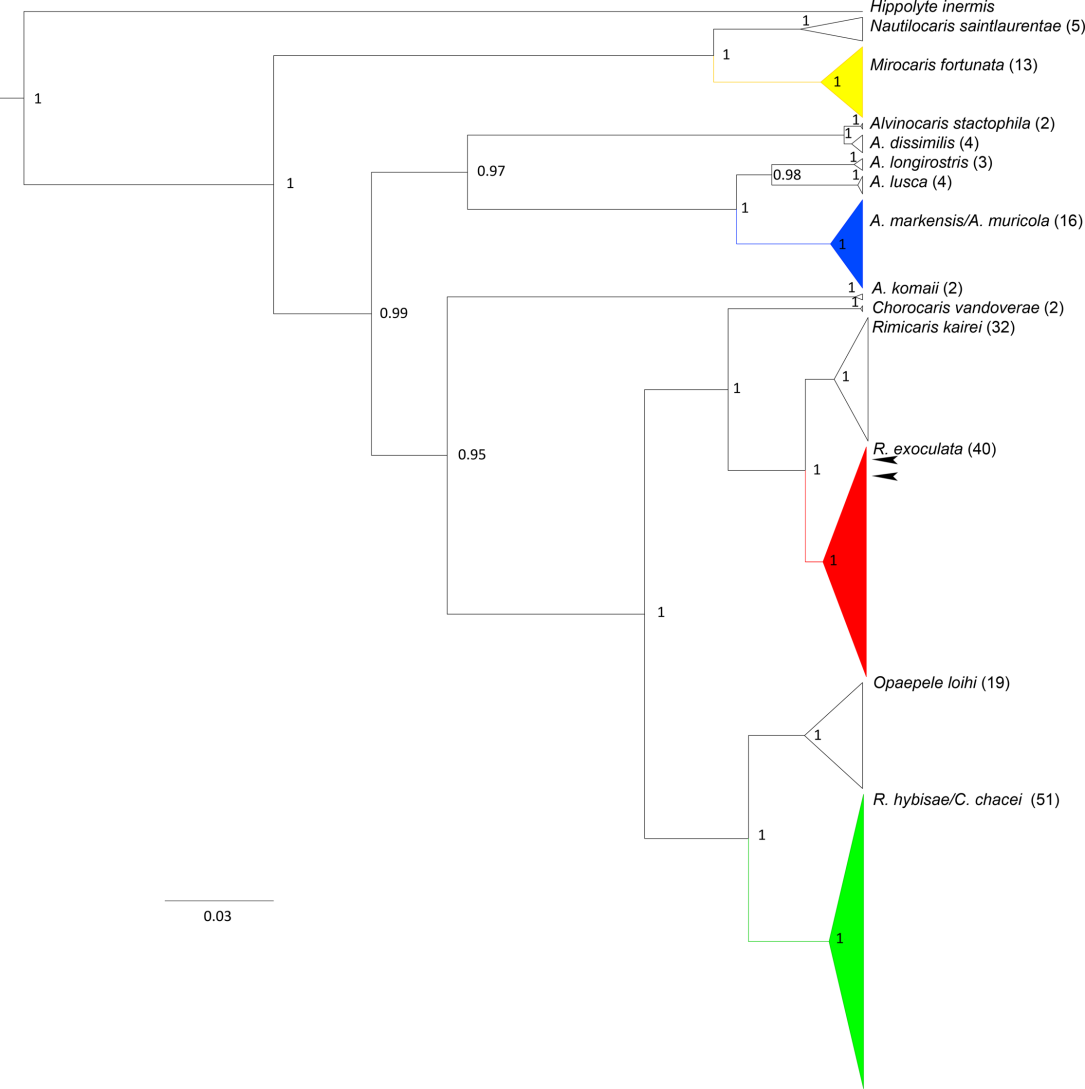
Phylogenetic relationships of Alvinocarididae shrimps based on the Bayesian Inference of COI gene using HYK + I + G evolutionary model. Species or monophyletic species-complex are cartooned and MAR species are in colors. Arrows shown the position of the larvae collected in the plankton samples of the MAR. Number of sequences in parentheses.

The phylogenetic tree obtained from Bayesian inferences with the COI gene shows three very divergent clades (Fig 8). The first clade consists of the genera *Mirocaris* and *Nautilocaris*, and includes the *N*. *saintlaurentae* hatching female considered in the present study. It is a sister group of the rest of Alvinocarididae. The second clade includes all species of *Alvinocaris* (6 spp.) except *A*. *komaii*. This group includes the sequence of Alvinocaridid larvae studied by Koyama *et al*. [10] which is affiliated with sequences of *Alvinocaris longirostris*. The third clade includes the genera *Rimicaris*, *Chorocaris* and *Opaepele*, in addition to one species of *Alvinocaris* (*A*. *komaii*). This 3-clade configuration is consistent with previous phylogenetic reconstruction based in COI gene [37], including the position of *A*. *komaii* [46]. The two species complexes formed by *Alvinocaris muricola/markensis* and by *Rimicaris chacei/hybisae*, both proposed by Teixeira *et al*. [6] and confirmed by Vereshchaka et al. [1] also appear in our phylogenetic analysis. This tree topology was expected since we used the same data as those presented by former authors, and it is further supported in our analysis which also includes a new sequences of *R*. *hybisae* obtained by Plouviez *et al*. [38]. All the species and species-complexes in our analysis are well supported by the posterior probabilities of the Bayesian Inference. The larvae collected with the larval pump at the TAG site on the MAR and identified from their morphology as *R*. *exoculata*, fall in our tree with the other *R*. *exoculata* samples. The large divergence with other alvinocaridid species from the MAR brings no doubt about the genetic confirmation of the morphological identification of these larvae.

### Larval traits along the DWCC

**Fig 9:**
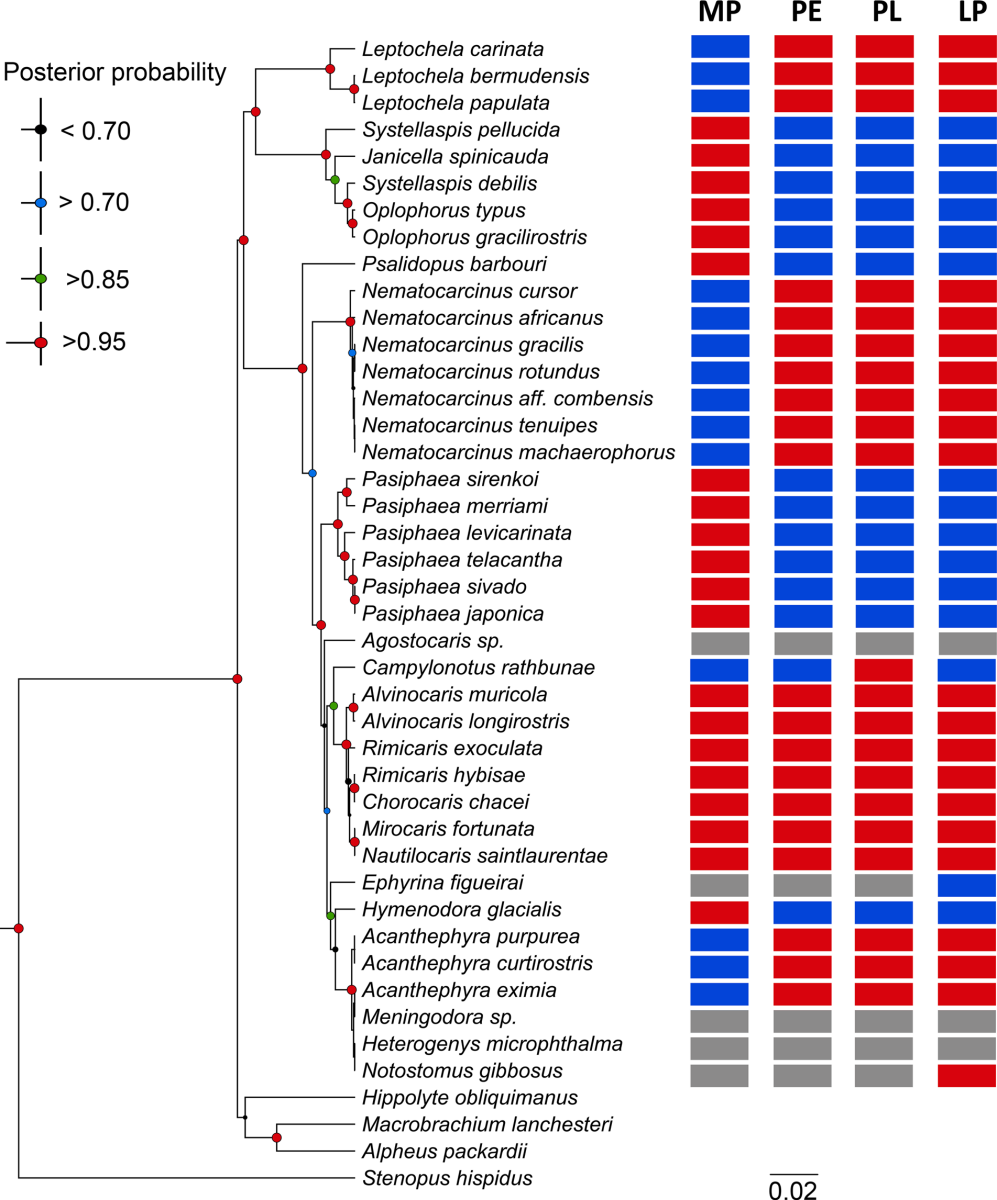
Phylogenetic relationships between Alvinocarididae and related families and distribution of larval traits along the tree. Phylogenetic reconstruction is based on the Bayesian Inference of 18S gene using HYK + G evolutionary model. Larval traits of first zoeal stage: MP, mouth parts developed (blue), non-developed (red); PE, pereiopods present (blue), absent (red); PL, pleopods present (blue), absent (red); LP, larval development abbreviated (blue), extended (red). Gray squares, non-information available.

Strong phylogenetic reconstructions of the DWCC based on several nuclear and mitochondrial loci are already published, but they do not necessarily include species with published information on larval traits. Here we selected species from different DWCC families with such knowledge as well as genetic information (most of the time 18S rRNA sequence) available. Our phylogenetic reconstruction of the DWCC based on the 18S gene (Fig 9) shows some differences with previous reconstructions [39–41], particularly in the position of Acanthephyridae and some families represented by single species (Campylonotidae, Psalidopodidae and Agostocarididae). These differences are attributed to the taxa coverage and locus selection. However the general topology of the tree is in agreement with some of the important phylogenetic features in the group previously suggested. Particularly, our tree also reflects the separation of Acanthephyridae *sensu stricto* from Ophoploridae [39,41,47], the polyphyletic status of Pasiphaeidae [39], and retains the monophyletic status of Nematocarcinidae [39,40] (although our analysis includes a single genus) and Alvinocarididae [1, 37,39–41,46–48]. Moreover in all genera with more than one species (except *Acanthephyra* and *Systellaspis*) the genetic relationship was closer within genus. Since we recover here the main trends previously proposed in broader analyses, we consider our tree suitable to make inferences about larval morphology and ecology.

Based on general traits of the first larval stage (mouth parts development and pereiopod or pleopod development), DWCC considered here could be separated in four main groups: 1: lack of pereiopod and pleopods, and developed mouth parts, 2: presence of pereiopod or pleopods and developed mouth parts, 3: presence of pereiopod or pleopods and undeveloped mouth parts, 4: lack of pereiopod and pleopods, and undeveloped mouth parts. The first group includes species from several families (Pasiphaeidae: *Leptochela*, Nematocarcinidae: *Nematocarcinus*, Acanthephyridae: *Acanthephyra*). Larval traits associated with planktotrophic and extended development are considered plesiomorphic due to their general distribution in decapod crustaceans in both shallow and deep-water habitats and their occurrence in the basal taxa of Caridea (Disciadidae and Rhynchocinetidae) according Bracken *et al*. [39] and Aznar-Cormano *et al*. [41]. The second group includes Campylonotidae. Facultative primary lecithotrophy is also present in this group [49]. The third group, with abbreviated larval development (hatching in advanced stage) and undeveloped mouth parts, typically exhibits lecithotrophy, which is found in several families (Ophophoridae, Pasiphaeidae: *Pasiphaea*, Acanthephyridae: *Hymenodora* and Psalidopodidae). The forth combination of traits is found only in Alvinocarididae.

## Discussion

### Comparison of larvae between alvinocaridid species

Early stages of Alvinocarididae exhibit particular features such as a notorious lack of development in mouth parts and few setation in the inner margin of the maxillipeds. This observation is not an artifact due to abnormally precocious hatching caused by recovery stress. The absence of a cover layer over the appendices and telson confirms that larvae analyzed here are at first zoeal stage instead of a prezoea stage [50,51] that could occur just after hatching. Moreover, the similarity between the larvae of *R*. *exoculata* obtained on board from hatching at atmospheric pressure or in pressurized chambers and in the plankton pump also demonstrates that the low degree of development of mouth parts is neither the product of an ontogenetic anomaly caused by the pre-hatching decompression nor abnormal early hatching of undeveloped larvae.

The absence of setation on the mouth parts (except for the scaphonagtite, and the tip of the endopod of the maxilla) and on the endopod margin of the maxillipeds, with the almost complete absence of spines on the carapace and abdomen bring a configuration very similar between the larvae herein studied. Spinulation of mouth parts shows intraspecific variation that overlaps interspecific variation. However *R*. *exoculata* can be separated from the other species by the presence of a spine and setulose setae on the distal section of the endopod of the maxilla and a small sub-ocular spine on the carapace, additional to the pterigostomian spine. The other species studied exhibit two spines at the tip of the endopod of the maxilla, or one spine and one thick setae (with few or no setulae), but only the pterigostomian spine on the carapace. In addition, the distal spine projected on the external margin of the endopod of the maxilla is usually larger in *R*. *exoculata* than in other species. *A*. *muricola* can be distinguished by the occurrence of a thick setae at the tip of the endopod of the maxilla, although this character showed some variation from small simple spine-like setae to large setae occasionally with few setulae (resembling *R*. *exoculata*). The intraspecific variation in spinulation completely overlaps between *M*. *fortunata* and *N*. *saintlaurentae*, limiting the distinction of larvae of the 2 species based on morphological characters (Table 2).

**Table 2:** Variation in larval structures in Alvinocaridid larvae.

	*R*. *exoculata*		*M*. *fortunata*		*N*. *saintlaurentae*		*A*. *muricola*	
	MF	Range	MF	Range	MF	Range	MF	Range
**Carapace**								
Subocular spine	p	ra-p	a		a		a	
**Antennule**								
Aesthetascs	4	3–5	4	3–5	4	3–5	4	4–5
Other spines or setae	0	0–1	1	0–1				
**Antenna**								
Basal spine ratio/endopod	0.6	0.4-.06	0.6	0.6–0.7	0.8	0.6–1.1	0.6	0.4–0.6
Endopod, Nbr of joints	2	1–2	2	1–2	2		2	
Spine in the last joint	l, sd	a-l, sd-d	s, d	a-s	s, sd	s-l	d, l	s-l
**Mandible**								
Nbr of spines	1	0-3s	0	0–2	0	0–2	0	0–1
**Maxillule**								
Nbr spines coxal endite	2	0–4	3	2–4	4	0–5	5	3–7
Nbr spines basal endite	2	2–3	3	3–6	2	1–4	3	1–3
Subdistal spine endopod	1	1–2	1		1	1–2	1	1–3
Nbr of distal spines	2	2–4	2		3	2–4	2	
**Maxilla**								
Spines lobe 1 coxal	5	3–8	6	5–6	5	4–8	5	4–8
Spines lobe 2 coxal	3	1–3	3	2–3	3	2–3	2	2–3
Spines lobe 1 basal	3	2–4	3	2–3	3	2–3	3	2–3
Spines lobe 2 basal	3	2–4	3	2–4	3	2–4	3	2–4
Spines lobe 1 endopod	3	1–3	3		3	1–3	3	1–3
Spines lobe 2 endopod	2	1–2	1	1–2	2	1–2	1	1–2
Inner distal projection	st		sp		sp		st	1–2
Size of inner distal projection	l	m-l	S		s		s	1–2, s-l
Outer distal spine	m	m-l	S		s	a-s	s	a-l
**Maxilliped 1**								
Endopod segmentation	5	5–6	4	4–6	6	i5-6	5	
Distal setae	2pd	1-2pd+ 0–1, s+ 1-3sp	2pd	2-3pd +1sp	3pd	2-3pd +1-2sp	1pd+1sp	1-2sp
Spinules inner margin	6	2–8	2	2–8	5	3–11	4	2–5
Spinules in the basis	2	0–8	4	0–4	3	0–5	3	0–4
Exopod segmentation	3		3		3	2–3	3	
Distal setae	3		3	1–3	3	2–3	3	2–3
Subdistal Setae	0	0–1	1	0–1	0	0–1	0–1	0–2
**Maxilliped 2**								
Endopod segmentation	6	5–6	i4	3–6	i6	3–6	6	5–6
Distal setae	2	1–3+0-1s+ 0-1sp	2	2–3+1sd, s	2	2–3+1sd, s	1	1–2+0-2s+1-2sp
Spinules inner margin	5	0–5	4	4–10	3	3–8	3	3–4
Spinules in the basis	0	0–3	0	0–3	1	0–4	3	2–3
Exopod segmentation	3		3	2–4	3	2–3	3	
Distal setae	3		3	3–4	3	2–3	0	
Subdistal Setae	0		0	0–3	1	0–1		
**Maxilliped 3**								
Endopod segmentation	5	5–6	i4	4–6	i6	3–6	6	5–6
Distal setae	2	1–2+ 0-1s+ 0-1sp	2	2–3+1sd	2	2–3+1sd	2 sp	0–2+0-1sp
Spinules inner margin	3	0–5	5	4–11	0	0–4	2	2–4
Spinules in the basis	0	0–2	0	0–2	0		3	2–3
Exopod segmentation	3		3		3		3	
Distal setae	3		3		3	2–4	3	
Subdistal Setae	0	0–2	0	0–2	1	1–2	0	

Columns show the mean or most frequent number or character (MF) and range. For each larval feature (only features with variation is considered). Keynote: a, absent; p, present; ra, rarely absent; s, small; m, medium; l, large; st, setae; sp, spine; d, distal; sd, subdistal; i, irregular. X-X denote a range and X+X denote additional structure (e.g. Distal setae: 0–2+0-1s = from 0 to 2 setae and from 0 to 1 small setae).

The larvae collected in plankton samples from Regab in the Gulf of Guinea are identified as first larval stage of *A*. *muricola*. These larvae, as well as those collected from a gravid female from the same site, showed a higher degree of morphological variations than zoea from the three other species herein studied. These variations were not related to growth and molting to the next larval stages since we did not observe changes in size or new structures (i.e. more setation or changes in size and shape in the appendices) as it would be expected after molting, as compared with the larvae obtained from onboard hatching. Adults and juveniles of *A*. *muricola* exhibit a wide range of morphological variations in some characters such as rostrum and carapace width [25]. *A*. *muricola* is the only alvinocaridid species known to inhabit the Congo basin cold seeps so far. However, this species is also found in the Barbados Prism, the Gulf of Mexico and the Blake Diapir [52]. Moreover, phylogenetic studies based on both mitochondrial (COI) and nuclear (18S rRNA) genes suggest that *A*. *muricola* and *A*. *markensis* from hydrothermal vents on the MAR are a single species [6]. *A*. *muricola* thus seems to be a morphologically plastic species, with plasticity also occurring in larvae as observed here, widely distributed and able to colonize different habitats, although morphological variability has not been explicitly related to specific locations or habitats so far.

Larvae collected with the plankton pump at the TAG vent field on the MAR were identified as first zoea stage of *R*. *exoculata* based on their external morphology. Both specimens examined show a tiny subocular spine and large setae at the tip of the endopod of the maxilla, which is consistent with the description in *R*. *exoculata*. This identification is also confirmed by COI barcode. The occurrence of ovigerous females bearing eggs close to hatching (pers. observation) explain the presence of early larval stages in the water column near the vent, which probably originates from TAG and have not yet dispersed away.

The close similarities between the larvae of *Rimicaris*, *Mirocaris*, *Nautilocaris* and *Alvinocaris* and their differences with other carideans (see next section), support the monophyletic status of Alvinocarididae previously suggested based in molecular phylogeny [37,39–41] and morphology, although genetic evidence suggests that the generic relationships within the family require a revision [1, 6,37]. Unfortunately, our taxa coverage and the overlapping of larval characters between the species herein studied preclude any approximation of within family phylogenetic relationship based on larval morphology.

### Morphology and larval biology of alvinocaridids

The absence of both masticatory processes in the mandible and setation in mouthparts that could participate in the process of capture and manipulation of food suggests that the alvinocaridid first larval stage is a non-feeding larva. This contrasts with previous hypothesis of planktotrophic larval nutrition in Alvinocarididae [9,11,18,53] based on indirect (egg size) evidence [11]. Although egg size in alvinocaridids is relatively smaller than in other species with lecithotrophic larvae [54], the accumulation of triacylgycerols, wax esters and monounsaturated fatty acids in eggs of alvinocaridid species [55] supports the hypothesis of primary lecithotrophy in this family. The occurrence of lipid storage in early stages is common in decapod larvae for standing eventual starvation or even lecithotrophy [20], but the suppression of feeding structures is rare. Lipid reserves in a lecithotrophic larvae or lacking of developed feeding structures has been documented previously in land crabs [56,57], symbiotic crabs [58], symmetric hermit crabs [59–61], galatheids [62,63], alpheid shrimps [64,65], freshwater shrimps (Atydae, [66]) and in some deep water Oplophoridae, Acanthephyridae and Pasiphaeidae [66, 67]. Larvae of these species also exhibit developed pleopods or pereiopods, which are common in late larvae, suggesting abbreviated development. These features are associated with restricted larval dispersal due to the short development period and larval or postlarval retention [56,58,64,68]. In alvinocaridids, although the early larval stage is lecithotrophic, the absence of pleopod and pereiopod does not support abbreviated development and genetic connectivity also suggest high dispersal [5,6,51]. Moreover, Koyama *et al*. [13] report that the early stage (zoea I) larvae of *Alvinocaris* sp. (which belong to *A*. *longirostris* according to our COI analysis) could survive for at least 74 days under laboratory conditions (atmospheric pressure, 4.5°C).

Related taxa (Pasiphaeidae) do not develop mouthparts during the larval stage and could survive without food source until the post-larvae [69], however these larvae also have abbreviated development that could be completed in 12 days (at 13°C). In alvinocaridids, the large amount of lipid reserve could be not enough to support the survivorship and growth in a complete extended larval development. According to Pond *et al*. [55] adults and eggs of *M*. *fortunata* have a similar lipid composition, dominated by monounsaturated and saturated fatty acids, lipids being transferred from the parent during vitellogenesis. However, between adults of both *R*. *exoculata* and *M*. *fortunata* and their respective juveniles, a shift in lipid composition occurs, with lipids in the juveniles dominated by polyunsaturated fatty acids and with different isotopic signature [55,70]. This suggests the accumulation of new lipid reserves before the recruitment to the vents, which could supply, at least partially the period between the recruitment, maturity and the acquisition of symbiotic bacteria [16]. Since the generation of new lipid reserves usually requires feeding, according to Pond *et al*. from a photosynthetic carbon origin [55,70], it is expected that the early lecithotrophic period would be followed by a feeding period during the larval development.

Although lipid composition of *R*. *exoculata*, *R*. *chacei*, *A*. *markensis* and *M*. *fortunata* at juvenile stage suggest feeding from photosynthetic source [55,70], similar to bathypelagic shrimps living close to hydrothermal vents in the MAR [71], it is still unclear which habitat the larval stages use. Post-larval stages of Alvinocarididae have been collected at a long distance from their potential origin (> 100 km) [9,70,72] in bathypelagic habitat between 1990–3060 m. Although Alvinocarididae early stages seem to tolerate large pressure variation, with larvae that can, in some cases, hatch and survive at atmospheric pressure ([12,13], present study), temperature tolerance may constrain the upper limit of the bathypelagic habitat [12]. An alternative hypothesis to explain the presence of lipids with photosynthetic isotopic signature in alvinocaridid shrimp is the feeding on particles descending to the aphotic zone, which are found near to the hydrothermal plumes [73] or in the open sea. At the present there is no direct evidence to support the occurrence of alvinocaridid larvae in the photic zone.

### Differences in larval morphology of alvinocaridid larvae with others caridean shrimps

General characters in the Alvinocarididae first larvae include tiny rostrum hidden between the eyes, pterisgostomial spine present, setation absent in mouth parts (except for scaphognathite and occasionally the tip of the endopod of the maxilla), mandible thump-like unarmed or with only 1–2 small spines, incisive and molar processes absent, maxilliped 1–3 similar in form and size, three large setae in the distal join of the exopod and 1–3 distal setae in the tip of the endopod, inner margin of endopod only with spinules. Since the present study is the first detailed description of early stage of alvinocaridid shrimps larvae, no other information is available yet for comparisons within the family.

In order to compare the larval morphology of Alvinocarididae with other carideans, it is important to consider their phylogenetic relationships. Although this family had been included in the Superfamily Bresilioidea [74], molecular evidence did not support monophyly at this level [39,41] or relationship between Alvinocarididae and Bresiliidae [41]. Phylogenetic relationships proposed by Tokuda *et al*. [75] suggest monophyly of Alvinocarididae with Palaemonidae, however general phylogenetic reconstruction of carideans using both mitochondrial and nuclear genes support the phylogenetic relationship of Alvinocarididae with Oplophoridae, Acanthephyridae, Nematocarcinidae, Pasiphaeidae [39–41], and also Agostocarididae, Psalidopodidae [39] and Campylonotidae [40]. Although there are evidences of polyphyly within Pasiphaeidae, all members tested of this family belong to the same general clade [39–41].

Related with a close morphological comparison of the larvae, Thatje *et al*. [76] highlighted the larval similarities between Campylonotidae and Oplophoridae *sensu lato* (Oplophoridae+Acantephyridae) based on the absence of external setae in maxillule, occurrence of four well developed endites on the maxilla and presence of exopods in all pereiopods. These structures of the maxillule and maxilla are described in the first larval stage of Oplophoridae, Acanthephyridae, Nematocarcinidae, Psalidopodidae and Campylonotidae [49,76–79], except for the occurrence of external setae in some Nematocarcinidae. Although setal interpretation is not possible for Psalidopodidae, due to description of late embryonic stage [79], both pairs of coxal and basal endites of the maxilla are present in this larva. Additionally, in three genera of Oplophoridae (*Systellaspis*, *Janicella* and *Oplophorus*), two Acanthephyridae (*Hymenodora* and *Acanthephyra*) and two Pasiphaeidae (*Pasiphaea* and *Parapasiphae*) a first larval stage with undeveloped mouth parts has been described [67,69,77]. Although the occurrence of pleopods at this stage for all previous taxa suggest also abbreviated larval development [66,77], which also had been suggested for Psalidopodidae [79,80]. The combination of lack of development of mandible and maxillule, the almost complete absence of setation at the inner margin of maxillule, maxilla and maxillipeds and the absence of pleopods all advocate for a new larval configuration in marine caridean shrimps.

Scattered distribution of the genera with lecithotrophic larvae or abbreviated development in the present phylogenetic reconstruction suggests that reduction of mouth parts development and abbreviated development evolved independently along major taxa. No pattern is observed comparing the distribution of the larval traits in the phylogenetic reconstructions proposed previously [39–41,47], although differences in the position of the taxa occurs between studies due to gene and taxa coverture. However, combinations of larval traits are consistent within families or monophyletic units (for Pasiphaeidae), except for *Hymenodora glacialis* and *Ephyrina figuerai*, which show different traits compared to other Acanthephyridae. Polyphyletic taxa, such as Oplophoridae (sensu lato) (Ophophoridae + Acanthephyridae) [39,41,47] and Pasiphaeidae [39], exhibit distinct larval trait combinations, which further support the genetic evidence that split them into monophyletic groups. Concerning Acanthephyridae, the species with abbreviated development are in a sister position of other Acanthephyridae with extended development, although the information about the larval traits in this group is incomplete. Larval traits seem to evolve through different events to the acquisition of the larval form, however these events are not observed at genera or species level of diversification, at least for most of the DWCC. The sequence of acquisition of different larval traits cannot be determined because the relationships between the monophyletic units are still not fully resolved, and there are discrepancies between the previous studies.

Although low variation in the larval traits is observed within the families (or monophyletic units) in the DWCC, other carideans show differences at this level. For instance, three distant and diverse monophyletic families as Alpheidae, Pandalidae and Atyidae [39–41] have species with planktotrophic and extended development but also species with lecithotrophic and abbreviated development [65,81,82]. This variation is associated with the distribution of the species in different habitats and the acquisition of different mechanisms for dispersal or for retention of the offspring. In alvinocaridids the restriction of the species to hydrothermal vents and cold seeps habitats and the common characteristics of these systems (deep waters, fragmented and widely distributed and dominance of bacterial chemosynthesis) could be related to the lack of diversification of larval traits.

## Conclusion

Alvinocaridid first larval stage is very distinctive from other decapod crustaceans because of the combination of undeveloped mouth parts and lack of pereiopods and pleopods, which suggest lecithotrophy but extended larval development. The larvae are very similar between the four genera and species studied, but minor morphological structures could be used for species identification. The larval traits observed in Alvinocarididae contrast with other larval models in decapod crustaceans, where lecithotrophy is associated to abbreviated development and some degree of larval retention. This model is consistent with a wide dispersal in the oligotrophic bathypelagic environment to colonize fragmented habitats such as hydrothermal vents and cold seeps. In the DWCC the scattered distribution of traits associated with lecithotrophic/planktotrophic and abbreviated/extended development suggests that they evolved independently in different combinations.

## Supporting Information

S1 Table Genebank references of COI sequences used in the present study. (PDF)

S2 Table Sequences (18S gene) included in the phylogenetic reconstruction and the larval traits of the species. The list includes information on larval traits of species not considered in the phylogenetic reconstruction, but used to make inferences on closely related species included in our phylogenetic reconstruction but without available data on larval traits (see methods).
(PDF)
