# Supplementary material for: Morphology of First Zoeal Stage of Four Genera of Alvinocaridid Shrimps from Hydrothermal Vents and Cold Seeps: Implications for Ecology, Larval Biology and Phylogeny
Source: PLoS One. 2015 Dec 28;10(12):e0144657. doi: 10.1371/journal.pone.0144657 (PMC4694104; doi:10.1371/journal.pone.0144657)
Supplement: S1 Table — (PDF) [file pone.0144657.s001.pdf]

S1 Table. **Genebank references of COI sequences used in the present study.**

Species	Genebank number	Reference
Hippolyte inermis (outgroup)	JF794740.1	[1]
Mirocaris fortunata	KT210460	Present study
	KT210450	Present study
	KT210451	Present study
	KT210452	Present study
	KT210453	Present study
	KT210454	Present study
	KT210455	Present study
	FJ769225.1	[2]
	FJ769226.1	[2]
	AF125430.1	[2]
	AF125431.1	[2]
	AF125432.1	[2]
	AF125433.1	[2]
Alvinocaris dissimilis	AB779491.1	Hiraoka,R. et al. (unpublished)
	AB779492.1	Hiraoka,R. et al. (unpublished)
	AB779493.1	Hiraoka,R. et al. (unpublished)
	AB779494.1	Hiraoka,R. et al. (unpublished)
Alvinocaris komaii	EU031816.1	[3]
	KP759373.1	[4]
Alvinocaris longirostris	AB222051.2	[5]
	AB222050.2	[5]
Alvinocaris lusca	AF125407.1	[2]
	AF125406.1	[2]
	AF125405.1	[2]
	AF125404.1	[2]
Alvinocaris markensis	KC840880.1	[6]
	KC840879.1	[6]
	KC840893.1	[6]
	KC840886.1	[6]
	KC840881.1	[6]
	KC840882.1	[6]
	KC840883.1	[6]
	KC840884.1	[6]
	KC840885.1	[6]
Alvinocaris muricola	KC840887.1	[6]
	KC840888.1	[6]
	KC840889.1	[6]
	KC840894.1	[6]
	KC840891.1	[6]
	KC840892.1	[6]
	KC840890.1	[6]
Alvinocaris stactophila	AF125410.1	[2]
	AF125411.1	[2]
Alvinocaris sp.	AB128829.1	[7]
Rimicaris chacei	KT210443	Present study

	KT210444	Present study
	KT210445	Present study
	KC840932.1	[6]
	KC840933.1	[6]
	KC840939.1	[6]
	KC840938.1	[6]
	KC840930.1	[6]
	KC840929.1	[6]
	KC840940.1	[6]
	KC840935.1	[6]
	KC840931.1	[6]
	KC840934.1	[6]
	KC840936.1	[6]
	KC840928.1	[6]
Rimicaris vandorvae	AF125417.1	[8]
	AF125418.1	[8]
Opaepele loihi	DQ328838.1	Jones et al. (unpublished)
	AF125437.1	[8]
	AF125436.1	[8]
	DQ328825.1	Jones et al. (unpublished)
	DQ328824.1	Jones et al. (unpublished)
	DQ328837.1	Jones et al. (unpublished)
	DQ328826.1	Jones et al. (unpublished)
	DQ328823.1	Jones et al. (unpublished)
	DQ328835.1	Jones et al. (unpublished)
	DQ328832.1	Jones et al. (unpublished)
	DQ328820.1	Jones et al. (unpublished)
	DQ328830.1	Jones et al. (unpublished)
	DQ328834.1	Jones et al. (unpublished)
	DQ328821.1	Jones et al. (unpublished)
	DQ328827.1	Jones et al. (unpublished)
	DQ328833.1	Jones et al. (unpublished)
	DQ328831.1	Jones et al. (unpublished)
	DQ328819.1	Jones et al. (unpublished)
Rimicaris exoculata	HM125956.1	[9]
	HM125918.1	[9]
	HM125927.1	[9]
	HM125935.1	[9]
	HM125950.1	[9]
	HM125949.1	[9]
	HM125937.1	[9]
	HM125926.1	[9]
	FN392999.1	[10]
	HM125911.1	[9]
	HM125910.1	[9]
	HM125921.1	[9]
	HM125943.1	[9]
	KT210447	Present study
	KT210446	Present study
	FN393004.1	[10]

	KT210448	Present study
	KT210449	Present study
	AF044057.1	[8]
	AF125419.1	[8]
	AF125403.1	[8]
	AF125402.1	[8]
	AF125401.1	[8]
	AF125398.1	[8]
	AF125399.1	[8]
	AF125400.1	[8]
	AF125420.1	[8]
	AF125440.1	[8]
	FN393000.1	[10]
	HM125925.1	[9]
	FN393005.1	[10]
	FN393007.1	[10]
	FN393006.1	[10]
	HM125922.1	[9]
	FN393001.1	[10]
	FN393003.1	[10]
	FN392996.1	[10]
	FN393002.1	[10]
	FN392998.1	[10]
	FN392997.1	[10]
Rimicaris hybisae	JN850607.1	[11]
	KJ566979.1	[12]
	KJ566987.1	[12]
	KJ566988.1	[12]
	KJ566974.1	[12]
	KJ566990.1	[12]
	KJ566971.1	[12]
	KJ566970.1	[12]
	KJ566992.1	[12]
	KJ566969.1	[12]
	KJ566975.1	[12]
	KJ566995.1	[12]
	KJ566980.1	[12]
	KJ566991.1	[12]
	KJ566981.1	[12]
	KJ567001.1	[12]
	KJ566997.1	[12]
	KJ566986.1	[12]
	KJ566976.1	[12]
	KJ566996.1	[12]
	KJ566989.1	[12]
	KJ566985.1	[12]
	KJ566968.1	[12]
	KJ566982.1	[12]
	KJ566977.1	[12]
	KJ566973.1	[12]

	KJ566978.1	[12]
	KJ566983.1	[12]
	KJ567003.1	[12]
	KJ567002.1	[12]
	KJ567000.1	[12]
	KJ566999.1	[12]
	KJ566998.1	[12]
	KJ566984.1	[12]
	KJ566972.1	[12]
Rimicaris kairei	AB813089.1	[13]
	AB813107.1	[13]
	AB813101.1	[13]
	AB813105.1	[13]
	AB813090.1	[13]
	AB813099.1	[13]
	AB813088.1	[13]
	AB813097.1	[13]
	AB813096.1	[13]
	AB813095.1	[13]
	AB813094.1	[13]
	AB813093.1	[13]
	AB813092.1	[13]
	AB813091.1	[13]
	AB813108.1	[13]
	AB813106.1	[13]
	AB813104.1	[13]
	AB813103.1	[13]
	AB813102.1	[13]
	AB813100.1	[13]
	AB813098.1	[13]
	AB813087.1	[13]
Nautilocaris sainlaurentae	KT223499	Present study
	KT223500	Present study
	KT223501	Present study
	NC_021971.1	[14]
	KF226726.1	[14]

References

1. Terosi M, Cuesta JA, Wehrtman IS, Mantelatto FL (2010) Revision of the larval morphology (Zoea I) of the family Hippolytidae (Decapoda Caridea), with a description of the first stages of the shrimp *Hippolyte obliquimanus* Dana, 1852. Zootaxa 2624: 49-66.
2. Bonnivard E, Catrice O, Ravaux J, Brown S, Higuet D (2009) Survey of genome size in 28 hydrothermal vent species covering 10 families. Genome 52: 524-536.
3. Zelnio KA, Hourdez S (2009) A New Species of Alvinocaris (Crustacea: Decapoda: Caridea: Alvinocarididae) from Hydrothermal Vents at The Lau Basin, Southwest Pacific, and a Key to The Species of Alvinocarididae. Proceedings of the Biological Society of Washington 122: 52-71.

4. Aznar-Cormano L, Brisset J, Chan T-Y, Corbari L, Puillandre N, et al. (2015) Hierarchical taxonomic sampling is a necessary but not sufficient condition for resolving inter-families relationships in Caridean decapods. Genetica 145: 195-205.
5. Tokuda G, Yamada A, Nakano K, Arita N, Yamasaki H (2006) Occurrence and recent long-distance dispersal of deep-sea hydrothermal vent shrimps. Biology Letters 2: 257-260.
6. Teixeira S, Olu K, Decker C, Cunha RL, Fuchs S, et al. (2013) High connectivity across the fragmented chemosynthetic ecosystems of the deep Atlantic Equatorial Belt: efficient dispersal mechanisms or questionable endemism? Molecular Ecology 22: 4663-4680.
7. Koyama S, Nagahama T, Ootsu N, Takayama T, Horii M, et al. (2005) Survival of deep-sea shrimp (*Alvinocaris* sp.) during decompression and larval hatching at atmospheric pressure. Marine Biotechnology 7: 272-278.
8. Shank TM, Black MB, Halanich KM, Lutz RA, Vrijenhoek RC (1999) Miocene radiation of the deep-sea hydrothermal vent shrimp (Caridea: Bresiliidae): Evidence from mithochondrial cytochrome oxidase subunit I. Molecular Phylogenetic and Evolution 13: 244-254.
9. Teixeira S, Cambon-Bonavita M-A, Serrão EA, Desbruyéres D, Arnaud-Haond S (2011) Recent population expansion and connectivity in the hydrothermal shrimp *Rimicaris exoculata* along the Mid-Atlantic Ridge. Journal of Biogeography 38: 564-574.
10. Petersen JM, Ramette A, Lott C, Cambon-Bonavita M-A, Zbinden M, et al. (2010) Dual symbiosis of the vent shrimp Rimicaris exoculata with filamentous gamma- and epsilonproteobacteria at four Mid-Atlantic Ridge hydrothermal vent fields. Environmental Microbiology 12: 2204-2218.
11. Nye V, Copley J, Plouviez S (2012) A new species of *Rimicaris* (Crustacea: Decapoda: Caridea: Alvinocarididae) from hydrothermal vent fields on the Mid-Cayman Spreading Centre, Caribbean. Journal of the Marine Biological Association of the United Kingdom 92: 1057-1072.
12. Plouviez S, Jacobson A, Wu M, Van Dover CL (2015) Characterization of vent fauna at the Mid-Cayman Spreading Center. Deep Sea Research Part I: Oceanographic Research Papers 97: 124-133.
13. Beedessee G, Watanabe H, Ogura T, Nemoto S, Yahagi T, et al. (2013) High Connectivity of Animal Populations in Deep-Sea Hydrothermal Vent Fields in the Central Indian Ridge Relevant to Its Geological Setting. PLoS ONE 8: e81570.
14. Kim S-J, Pak SJ, Ju S-J (2013) Mitochondrial genome of the hydrothermal vent shrimp *Nautilocaris saintlaurentae* (Crustacea: Caridea: Alvinocarididae). Mitochondrial DNA 0: 1-2.
