# Supplementary material for: Morphology of First Zoeal Stage of Four Genera of Alvinocaridid Shrimps from Hydrothermal Vents and Cold Seeps: Implications for Ecology, Larval Biology and Phylogeny
Source: PLoS One. 2015 Dec 28;10(12):e0144657. doi: 10.1371/journal.pone.0144657 (PMC4694104; doi:10.1371/journal.pone.0144657)
Supplement: S2 Table — The list includes information on larval traits of species not considered in the phylogenetic reconstruction, but used to make inferences on closely related species included in our phylogenetic reconstruction but without available data on larval traits (see methods). (PDF) [file pone.0144657.s002.pdf]

S2 Table. **Sequences (18S gene) included in the phylogenetic reconstruction and the larval traits of the species.** The list includes information on larval traits of species not considered in the phylogenetic reconstruction, but used to make inferences on closely related species included in our phylogenetic reconstruction but without available data on larval traits (see methods).

	18S		Larval Morphology	General larval traits (first zoea)				
SPECIES	Source	Genbank	Source	Mouth parts development	Trophic status	Pereiopods	Pleopods	Type of development
PASIAPHAEIDAE								
Leptochela bermudensis	[1]	EU868785	[2,3]	Developed	Planktotrophic	Absent	Absent	Extended
L. papulata	[1]	EU868784						
L. carinata	[1]	EU868786						
L. gracilis			[4]	Developed	Planktotrophic	Absent	Absent	Extended
Pasiphaea japonica	[5]	JF346260	[6]	Undevelodep	Primary lecitotrophy	Present	Present	Abbreviated
P. merriami	[1]	EU868796						
P. levicarinata	[5]	JF346261						
P. sirenkoi	[7]	KP725823						
P. sivado	[7]	KP725826	[8]	Undevelodep	Primary lecitotrophy	Present	Present	Abbreviated
P. tarda			[3,8]	Undevelodep	Primary lecitotrophy	Present	Present	Abbreviated
P. telacantha	[7]	KP725828						
Parapasiphae sulcatifrons			[3,8]	Undevelodep	Primary lecitotrophy	Present	Present	Abbreviated
OPLOPHOIDAE								
Janicella spinicauda	[7]	KP075856.1	[3]	Undevelodep	Primary lecitotrophy	Present	Present	Abbreviated
Oplophorus typus	[9]	GQ131929.1	[3]	Undevelodep	Primary lecitotrophy	Present	Present	Abbreviated
O. gracilirostris	[9]	GQ131928	[3]	Undevelodep	Primary lecitotrophy	Present	Present	Abbreviated
O. spinosus			[10]	Undevelodep	Primary lecitotrophy	Present	Present	Abbreviated
Systellapis debilis	[1]	EU868775	[10]	Undevelodep	Primary lecitotrophy	Present	Present	Abbreviated
S. pellucida	[5]	JF346250						
ACANTHEPHYRIDAE								
A. cutirostris	[1]	EU868769						

A. brevirostris			[11]	Developed	Planktotrophic	Absent	Absent	Extended
A. purpurea	[1]	EU868770	[10,12]	Developed	Planktotrophic	Absent	Absent	Extended
A. eximia	[9]	GQ131916						
A. acutifrons			[12]	Developed	Planktotrophic	Absent	Absent	Extended
A. stylorostrata			[12]	Developed	Planktotrophic	Absent	Absent	Extended
Hymenodora glacialis	[9]	GQ131915	[3,13]	Undevelodep	Primary lecitotrophy	Present	Present	Abbreviated
Ephyrina figuerai	[14]	AM087654	[3]					Abbreviated
Meningodora sp.	[9]	GQ131931						
Notostomus gibbosus	[9]	GQ131917	[10]					Extended
Heterogenys microphthalma	[9]	GQ131932						
NEMATOCARCINIDAE								
Nematocarcinus africanus	[7]	KP725769						
N. lanceopes			[15]	Developed	Planktotrophic	Absent	Absent	Extended
N. cursor	[1]	U868766						
N. rotundus	[1]	EU868765						
N. gracilis	[5]	JF346240						
N. tenuipes	[5]	JF346248						
N. aff combensis	[5]	JF346238						
N. longirostris			[15]	Developed	Planktotrophic	Absent	Absent	Extended
AGOSTOCARIDAE								
Agostocaris sp.	[1]	EU868716						
PSALIDOPODIDAE								
Psalidopus barbouri	[1]	EU868804	[16]*	Undevelodep	Primary lecitotrophy	Present	Present	Abbreviated
CAMPYLONOTIDAE								
Campylonotus rathbunae	[5]	JF346246	[17]	Developed	Facultative lecitotrophy	Present	Absent	Abbreviated
C. vagans			[18,19]	Developed	Facultative lecitotrophy	Present	Absent	Abbreviated

C. capensis			[18]	Developed	Facultative lecitotrophy	Present	Absent	Abbreviated
C. semistriatus			[18]	Developed	Facultative lecitotrophy	Present	Absent	Abbreviated
ALVINOCARIDIDAE								
Alvinocaris longirostris	[5]	JF346247	[20]**			Absent	Absent	Extended
A. muricola	[1]	EU868717	this study	Undevelodep	Primary lecitotrophy	Absent	Absent	Extended
Rimicaris exoculata	[14]	AM087652	this study	Undevelodep	Primary lecitotrophy	Absent	Absent	Extended
R. hybisae	this study	KT210459	[21]***	Undevelodep	Primary lecitotrophy	Absent	Absent	Extended
R. chacei	this study	KT210456						
Mirocaris fortunata	this study	KT210458	this study	Undevelodep	Primary lecitotrophy	Absent	Absent	Extended
Nautilocaris saintlaurentae	this study	KT210457	this study	Undevelodep	Primary lecitotrophy	Absent	Absent	Extended
Outgroups								
Stenopodidae								
Stenopus hispidus	[5]	JF346253						
Hippolytidae								
Hippolyte obliquimanus	[1]	EU868752						
Alpheidae								
Alpheus packardii	[1]	EU868720.1						
Palaemonidae								
Macrobrachium lanchesteri	[7]	KP725756.1						

*Late embryo

**Analysis of COI sequence of *Alvinocaris* sp. submitted by Koyama *et al.* [20] suggest that the species belong to *A. longirostris* (see COI analysis in the present study). The mouth parts of the larvae are not shown in [20], we assume that they are similar than *A. muricola.* Other traits are observed from the illustration

***The mouth parts of the larvae are not shown in [21], we assume that they are similar than *R. exoculata.* Other traits are observed from the illustration.

References

1. Bracken HD, De Grave S, Felder D (2009) Phylogeny of the infraorder Caridea based on mitochondrial and nuclear genes (Crustacea: Decapoda). In: Martin JW, Crandal KA, Felder D, editors. Decapod Crustacean Phylogenetics: CRC Press. pp. 281-305.
2. Gurney R (1936) Notes on some Decapod Crustacea of Bermuda.—III.-V. Proceedings of the Zoological Society of London 106: 619-630.
3. Gurney R (1942) Larvae of decapod Crustacea. Royal Society of London 129: 1-306.
4. Sekiguchi H (1980) Larvae of *Leptochela gracilis* Stimpson (Decapoda: Natantia: Pasiphaeidae). Proceedings of the Japanase Society of Systematic Zoology 18: 30-46.
5. Li CP, De Grave S, Chan T-Y, Lei HC, Chu KH (2011) Molecular systematics of caridean shrimps based on five nuclear genes: Implications for superfamily classification. Zoologischer Anzeiger 250: 270-279.
6. Nanjo N, Konishi K (2009) Complete larval development of the Japanese glass shrimp *Pasiphaea japonica* Omori, 1976 (Decapoda: Pasiphaeidae) under laboratory conditions. Crustacean Research 38: 77-89.
7. Aznar-Cormano L, Brisset J, Chan T-Y, Corbari L, Puillandre N, et al. (2015) Hierarchical taxonomic sampling is a necessary but not sufficient condition for resolving inter-families relationships in Caridean decapods. Genetica 145: 195-205.
8. Williamson DI (1960) Larval stages of *Pasiphaea sivado* and some other Pasiphaeidae (Decapoda). Crustaceana 1: 331-341.
9. Chan T, Lei HC, Li CP, Chu KH (2010) Phylogenetic analysis using rDNA reveals polyphyly of Oplophoridae (Decapoda: Caridea). Invertebrate Systematics 24: 172-181.
10. Gurney R, Lebour MV (1941) On the Larvae of certain Crustacea Macrura, mainly from Bermuda. Journal of the Linnean Society of London, Zoology 41: 89-181.
11. Hendrickx M, Garcia-Guerrero M (2007) Description of the first zoea of *Acanthephyra brevicarinata* Hanamura, 1984 (Caridea: Oplophoridae) from the deep water of the Gulf of Mexico. Contributions of the Study of Easthern Pacific Crustaceans 4: 31-36.
12. Herring PJ (1967) Observations on the early larvae of three species of Acanthephyra (Crustacea, Decapoda, Caridea). Deep Sea Research and Oceanographic Abstracts 14: 325-329.
13. Stephensen K (1935) Crustacea Decapoda. The Godthaab Expedition 1928. Medd Granl 80: 1-94.
14. Leignel V, Van Wormhoudt A, Buit QT, Ravallec R, LAulier M Molecular phylogenetic relationships between deep-sea shrimp families : Analysis based on rDNA and mtDNA data. Unpublished.
15. Thatje S, Bacardit R, Arntz W (2005) Larvae of the deep-sea Nematocarcinidae (Crustacea: Decapoda: Caridea) from the Southern Ocean. Polar Biology 28: 290-302.
16. Martin JW (1985) A late embryo of the deep water shrimp *Psalidopus barbourdi* Chace, 1939 (Decapoda: Caridea). Crustaceana 59: 299-302.

17. Pike RB, Williamson DI (1966) First zoeal stage of *Campylonotus rathbunae* Schmitt and its bearing on the systematical position of the Campylonotidae 5Decapoda, Caridea). Transactions of the Royal Sociaety of New Zealand, Zoology 7: 209-213.
18. Thatje S, Bacardit R, Romero MC, Tapella F, Lovrich GA (2001) Description and key to the zoeal stages of the Campylonotidae (Decapoda: Caridea) from the magellan region. Journal of Crustacean Biology 21: 492-505.
19. Thatje S, Lovrich GA, Anger K (2004) Egg production, hatching rates, and abbreviated larval development of *Campylonotus vagans* Bate, 1888 (Crustacea: Deacapoda: Caridea), in subantartic waters. Journal of Experimental Marine Biology and Ecology 301: 15-27.
20. Koyama S, Nagahama T, Ootsu N, Takayama T, Horii M, et al. (2005) Survival of deep-sea shrimp (*Alvinocaris* sp.) during decompression and larval hatching at atmospheric pressure. Marine Biotechnology 7: 272-278.
21. Nye V, Copley JT, Tyler PA (2013) Spatial Variation in the Population Structure and Reproductive Biology of *Rimicaris hybisae* (Caridea: Alvinocarididae) at Hydrothermal Vents on the Mid-Cayman Spreading Centre. PLoS ONE 8: e60319.
